# Flubendazole induces mitochondrial dysfunction and DRP1-mediated mitophagy by targeting EVA1A in breast cancer

**DOI:** 10.1038/s41419-022-04823-8

**Published:** 2022-04-19

**Authors:** Yongqi Zhen, Zhaoxin Yuan, Jiahui Zhang, Yao Chen, Yuning Fu, Yi Liu, Leilei Fu, Lan Zhang, Xian-Li Zhou

**Affiliations:** 1 Sichuan Engineering Research Center for Biomimetic Synthesis of Natural Drugs, School of Life Science and Engineering, Southwest Jiaotong University, 610031 Chengdu, China; 2 Key Laboratory of Advanced Technologies of Materials, Ministry of Education, Southwest Jiaotong University, 610031 Chengdu, China

**Keywords:** Breast cancer, Pharmacology

## Abstract

Breast cancer is still one of the most common malignancies worldwide and remains a major clinical challenge. We previously reported that the anthelmintic drug flubendazole induced autophagy and apoptosis via upregulation of eva-1 homolog A (EVA1A) in triple-negative breast cancer (TNBC) and was repurposed as a novel anti-tumor agent. However, the detailed underlying mechanisms remain unclear and need further investigation. Here, we found that flubendazole impairs the permeability of the mitochondrial outer membrane and mitochondrial function in breast cancer. Meanwhile, flubendazole increased dynamin-related protein (DRP1) expression, leading to the accumulation of PTEN induced putative kinase 1 (PINK1) and subsequent mitochondrial translocation of Parkin, thereby promoting excessive mitophagy. The resultant excessive mitophagy contributed to mitochondrial damage and dysfunction induced by flubendazole, thus inhibiting breast cancer cells proliferation and migration. Moreover, we demonstrated that excessive DRP1-mediated mitophagy played a critical role in response to the anti-tumor effects of EVA1A in breast cancer. Taken together, our results provide new insights into the molecular mechanisms in relation to the anti-tumor activities of flubendazole, and may be conducive to its rational use in potential clinical applications.

## Graphical abstract

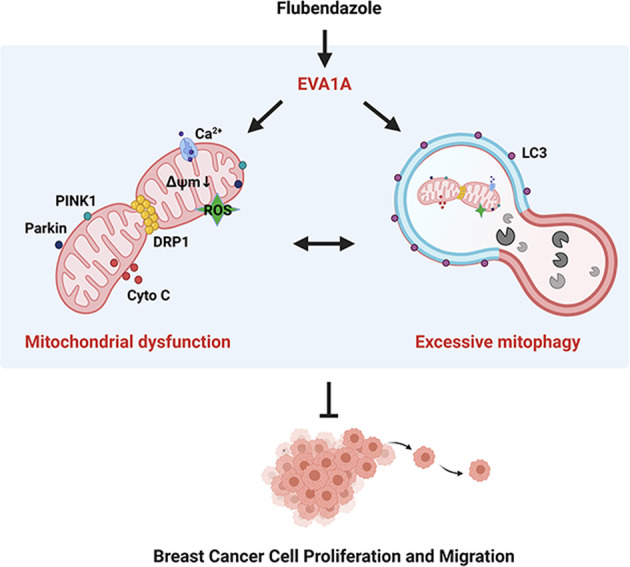

## Introduction

According to decades of epidemiological and clinical research, breast cancer incidence continues to rise and is still the most common malignancy worldwide among women [1]. Based upon the presence or absence of molecular markers for estrogen or progesterone receptors (ER or PR) and human epidermal growth factor 2 (HER2), breast cancer can be divided into three crucial subtypes: hormone receptor-positive, HER2-positive and triple-negative breast cancer [2]. Surgery, radiation, and endocrine therapy remain essential cornerstones of breast cancer therapy [3]. In addition, neoadjuvant therapy, including chemotherapy with targeted agents, has been widely used in breast cancer characterized by high recurrence and metastasis [4]. Unfortunately, most patients soon acquired resistance to these treatments and relapsed [5, 6]. Thus, new therapeutic agents are imperative to improve the prognosis of breast cancer patients.

Mitochondria are crucial organelles for bioenergetic, biosynthetic, cellular homeostasis and signal transduction in mammals [7]. Mitochondria also play a central role in regulating cell death, including apoptosis, necroptosis, pyroptosis and ferroptosis [7]. For example, the permeability of the outer mitochondrial membrane changes during mitochondrial-dependent apoptosis, accompanied by the release of soluble proteins such as cytochrome c (Cyto C) and subsequent caspase activation [8]. Numerous studies have shown that the alteration of mitochondrial function affects tumorigenesis, progression, and resistance to therapy, including the biogenesis and turnover of mitochondria, fission and fusion dynamics, cell death regulation, oxidative stress regulation, metabolism and bioenergetics [9, 10]. Furthermore, damaged mitochondria can be delivered to lysosomes for degradation through mitophagy, a selective autophagic process, to maintain mitochondrial homeostasis [11, 12]. However, in addition to the pro-survival mechanism, excessive or persistent mitophagy will undermine the health of mitochondria in tumorigenesis and metastasis, ultimately leading to autophagic cell death [13, 14]. Moreover, a growing body of research has shown that defected or impaired mitophagy could lead to pathological conditions. For example, BRCA1 deficiency impairs stress-induced mitophagy and triggers inflammasome activation, creating a tumor-associated microenvironment, facilitating tumor proliferation and metastasis [15]. Therefore, dissection of the mechanism underlying the dual role of mitophagy is crucial for exploiting mitophagy as a therapeutic approach in cancer treatment.

Drug repurposing (also called drug repositioning, reprofiling or re-tasking) is a strategy for identifying new uses for approved or investigational drugs, saving overall drug development costs and development time [16]. Compared with the development of new drugs, the most crucial advantage of drug repurposing is to significantly reduce the risk of failure, although it has great opportunities and is serendipitous [17, 18]. For example, thalidomide is active against advanced myeloma [19], and aspirin helps prevent cardiovascular disease and colorectal cancer [20]. The FDA approved Raloxifene in 2007 to treat osteoporosis also affects invasive breast cancer [21]. Growing evidence suggests that flubendazole, a broad-spectrum anthelmintic drug, alone or combined with other agents, has significant efficacy in various tumors, including breast cancer, and has been repurposed as a promising anti-cancer agent [22–24]. We have previously identified that flubendazole could induce autophagic cell death involved with ROS production [25]. Moreover, flubendazole could regulate autophagy and apoptosis via targeting EVA1A, thus affecting TNBC proliferation and migration [26]. However, the detailed underlying mechanisms of EVA1A remain unclear and need further investigation in breast cancer treatment with flubendazole.

In this study, we investigated the role of flubendazole-induced mitophagy in mitochondrial function and anti-cancer effects and elucidated the mechanism of DRP1-mediated mitophagy via targeting EVA1A. Moreover, we demonstrated that excessive DRP1-mediated mitophagy is characterized as a critical event in response to the anti-tumor effects of EVA1A. Together, these findings shed a novel mechanism of flubendazole against breast cancer, focusing on the mitochondrial dysfunction and DRP1-mediated mitophagy in breast cancer via targeting EVA1A.

## Results

### Flubendazole impairs the permeability of the mitochondrial outer membrane and induces mitochondrial dysfunction in breast cancer

**Fig. 1:**
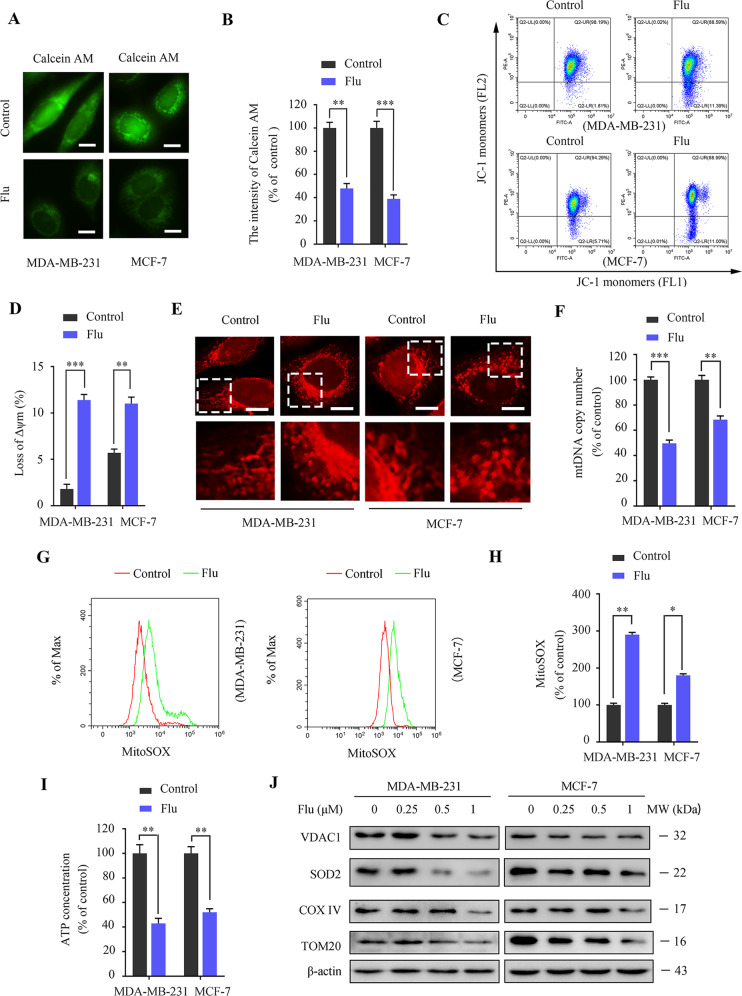
Flubendazole impairs the permeability of the mitochondrial outer membrane and induces mitochondrial dysfunction in MDA-MB-231 and MCF-7 cells. **A**, **B** MDA-MB-231 and MCF-7 cells were treated with or without flubendazole (0.5 μM) for 24 h. A fluorescence microscope evaluated the intensity of Calcein AM. Representative images and quantification of Calcein AM were shown. Scale bar, 5 µm. **C**, **D** Flow cytometric analysis and quantification of mitochondrial membrane potential changes in MDA-MB-231 and MCF-7 cells treated with or without flubendazole (0.5 μM) for 24 h. **E** Mitochondria were stained with MitoTracker^TM^ Deep Red FM probes for 30 min and observed with a confocal microscope. Representative images of mitochondrial morphology were shown. Scale bar, 5 µm. **F** RT-qPCR analysis of mitochondrial DNA copies in MDA-MB-231 and MCF-7 cells. **G**, **H** Mitochondria were stained with MitoSOX^TM^ Red FM for 30 min, and mitochondrial ROS accumulation was analyzed by flow cytometry. **I** ATP content measurement in MDA-MB-231 and MCF-7 cells treated with or without flubendazole (0.5 μM) for 24 h. **J** Immunoblotting analysis of VDAC1, SOD2, COX IV, TOM20 expression in MDA-MB-231 and MCF-7 cells treated with the indicated concentration of flubendazole for 24 h. β-actin was used as the loading control. Data represent mean ± SD. *P < 0.05, **P < 0.01, ***P < 0.001. Statistical significance compared with respective control groups (all *P*-values were obtained by one-way ANOVA).

Flubendazole-induced apoptosis was dependent on caspase activation in breast cancer [23, 24], and we also determined that flubendazole can up-regulate the pro-apoptotic protein Bax and downregulate the anti-apoptotic protein Bcl-2 in TNBC [26]. However, the subsequent effects of Bax and Bcl-2 on the permeability of the mitochondrial outer membrane need to be further confirmed in breast cancer treatment with flubendazole, which leads to the dissipation of mitochondrial membrane potential and provides a channel for the release of mitochondrial intermembrane space proteins (most importantly, Cyto C) [27]. Thus, we first evaluated the effects of flubendazole on the mitochondrial permeability transition pore (mPTP) opening in MDA-MB-231 and MCF-7 cells. Flubendazole treatment resulted in a significant decrease in the fluorescence intensity of Calcein AM compared with the control group, which indicates an increased rate of mPTP opening (Fig. 1A, B). Moreover, flubendazole-treated MDA-MB-231 and MCF-7 cells showed a decline in mitochondrial membrane potential as determined by the uptake of JC-1 (Fig. 1C, D). Then, we evaluated the effects of flubendazole on the release of Cyto C from mitochondria into the cytosol in MDA-MB-231 and MCF-7 cells. Western blot analysis revealed that flubendazole dose-dependently increased the content of Cyto C in the cytoplasm but decreased in a dose-dependently in mitochondria (Fig. S1A). An immunofluorescence assay also confirms that Cyto C was released from mitochondria into the cytosol in response to flubendazole treatment (Fig. S1B, C). These findings indicate that flubendazole impairs the permeability of the mitochondrial outer membrane in breast cancer.

Mitochondrial outer membrane permeability is often accompanied by damage to the mitochondria include morphological changes and dysfunction [28]. Then, we evaluated the morphology, quantity, and function of mitochondria in MDA-MB-231 and MCF-7 cells to ascertain the mitochondrial alterations in response to flubendazole treatment. The MDA-MB-231 and MCF-7 cells were stained with the MitoTracker^TM^ Deep Red FM probe. Compared with the control group, a significant increase number of mitochondria with ring-shaped structures in flubendazole-treated MDA-MB-231 and MCF-7 cells was observed, suggesting the occurrence of mitochondrial fission or even fragmentation (Fig. 1E). Likewise, we determined the relative mitochondrial number by quantifying the mitochondrial DNA copy number. As a result, flubendazole treatment reduced the mtDNA copy number, suggesting a decrease in the number of mitochondria (Fig. 1F). Meanwhile, we found that the mitochondrial function was aberrant, as shown by decreased ATP levels and increased superoxide in MDA-MB-231 and MCF-7 cells induced by flubendazole (Fig. 1G–I). Additionally, the expression levels of mitochondrial components such as SOD2, VDAC1, COX IV, and TOM20 were downregulated in flubendazole-treated MDA-MB-231 and MCF-7 cells (Fig. 1J). Together, these results suggest that flubendazole induces morphological changes and impairs mitochondrial function in breast cancer cells.

### Flubendazole promotes mitophagy via PINK1/Parkin signaling in breast cancer

**Fig. 2:**
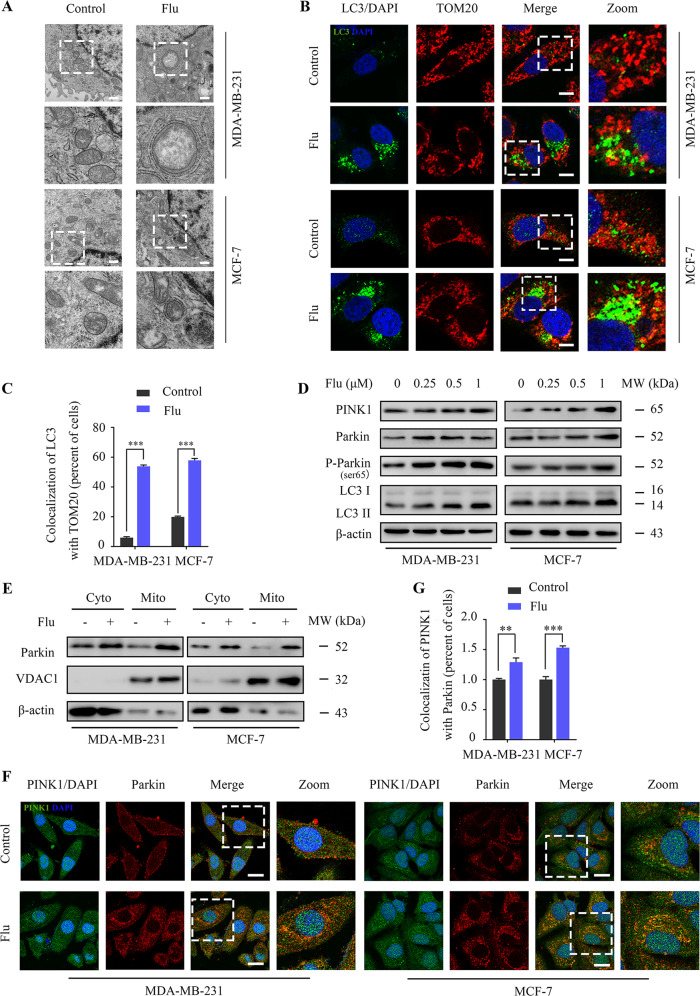
Flubendazole promotes mitophagy via PINK1/Parkin signaling in MDA-MB-231 and MCF-7 cells. **A** MDA-MB-231 and MCF-7 cells were treated with or without flubendazole (0.5 μM) for 24 h. The images were captured with a transmission electron microscope. Scale bar, 500 nm. **B**, **C** The autophagosomes are labeled by LC3 (green fluorescence) protein and the mitochondria are labeled by TOM20 (red fluorescence) protein. The number of co-localized LC3 and TOM20 was quantified. Scale bar, 5 µm. **D** Immunoblotting of PINK1, Parkin, p-Parkin^ser65^ and LC3 in MDA-MB-231 and MCF-7 cells treated with the indicated concentrations of flubendazole for 24 h. β-actin was used as the loading control. **E** Immunoblotting of Parkin in the cytosolic and mitochondrial fractions of MDA-MB-231 and MCF-7 cells treated with or without Flubendazole (0.5 μM) for 24 h. β-actin (cytoplasmic fraction) and VDAC1 (mitochondrial fraction) were used as the loading controls. **F**, **G** Colocalization of PINK1 (green fluorescence) protein and Parkin (red fluorescence) protein in MDA-MB-231 and MCF-7 cells following flubendazole (0.5 μM, 24 h) treatment. The number of co-localized PINK1 and Parkin was quantified. Scale bar, 10 µm. Data represent mean ± SD. *P < 0.05, **P < 0.01, ***P < 0.001. Statistical significance compared with respective control groups (all *P*-values were obtained by one-way ANOVA).

Mitophagy is a form of clearing damaged or malfunctioning mitochondria, which is essential for the quality and maintenance of mitochondria [29]. To determine whether flubendazole induced mitophagy in breast cancer, we observed subcellular organelles by transmission electron microscopy and found that there are incompletely degraded mitochondrial cristae in autolysosomes (Fig. 2A). In addition, flubendazole significantly increased colocalization of the autophagosome with the mitochondria, as evidenced by the merged fluorescent signaling of LC3 and TOM20 (Fig. 2B, C). Since many molecules were involved in autophagosome formation, and the PINK1/Parkin pathway is one of the critical signaling pathways that mediate mitophagy in mammalian cells [30]. Thus, we detected the expression levels of PINK1 and Parkin after flubendazole treatment. As shown, flubendazole treatment increased the expression of PINK1, Parkin, p-Parkin^ser65^ and LC3 in MDA-MB-231 and MCF-7 cells (Fig. 2D). As the translocation of Parkin to mitochondria is a hallmark of mitophagy [11], we then examined mitochondria translocation of Parkin in flubendazole-treated cells by analyzing cellular fractionations. As expected, we observed enriched Parkin in the mitochondria fraction in flubendazole-treated MDA-MB-231 and MCF-7 cells (Fig. 2E). Consistently, these results were further supported by the increased level of colocalization of PINK1 and Parkin in flubendazole-treated MDA-MB-231 and MCF-7 cells (Fig. 2F, G). Altogether, these data suggest that flubendazole stimulates mitophagy via PINK1/Parkin signaling in breast cancer.

### Flubendazole induces mitochondrial dysfunction by DRP1-mediated mitophagy in breast cancer

**Fig. 3:**
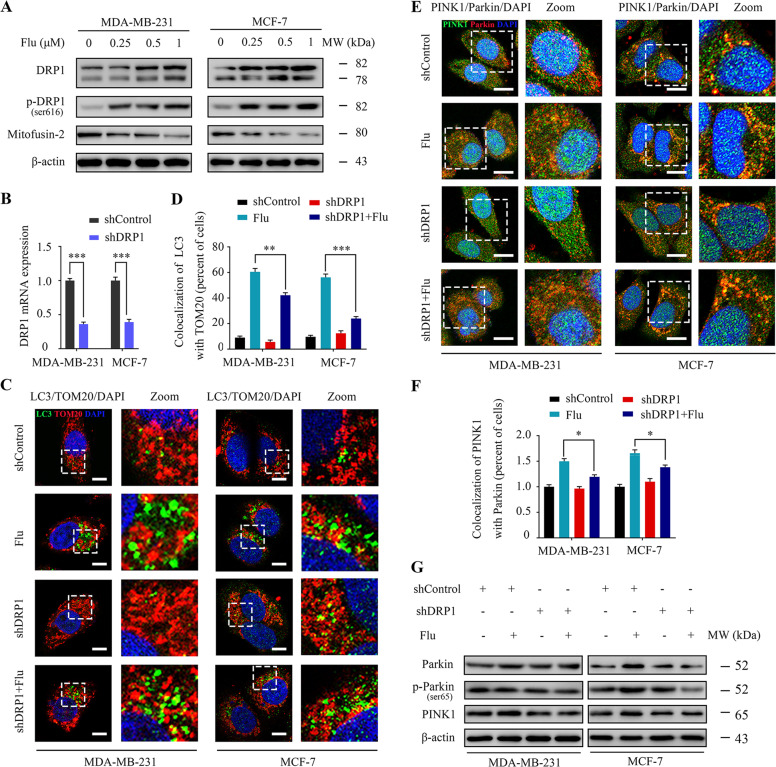
Flubendazole induces DRP1-mediated mitophagy in MDA-MB-231 and MCF-7 cells. **A** Immunoblotting of DRP1, p-DRP1^ser616^ and Mitofusin-2 in MDA-MB-231 and MCF-7 cells treated with the indicated concentrations of flubendazole for 24 h. β-actin was used as the loading control. **B** *DRP1* mRNA expression in MDA-MB-231 and MCF-7 cells was analyzed by RT-qPCR. **C**, **D** MDA-MB-231 and MCF-7 cells were transfected with negative-control or *DRP1* shRNA for 24 h, respectively. After treatment with or without flubendazole (0.5 μM) for 24 h. The autophagosomes are labeled by LC3 (green fluorescence) protein, and the mitochondria are labeled by TOM20 (red fluorescence) protein. The number of co-localized LC3 and TOM20 was quantified. Scale bar, 5 µm. **E**, **F** Colocalization of PINK1 (green fluorescence) protein and Parkin (red fluorescence) protein in MDA-MB-231 and MCF-7 cells following flubendazole (0.5 μM, 24 h) treatment. The number of co-localized PINK1 and Parkin was quantified. Scale bar, 10 µm. **G** Immunoblotting of Parkin, p-Parkin^ser65^ and PINK1 expression. β-actin was measured as the loading control. Data represent mean ± SD. *P < 0.05, **P < 0.01, ***P < 0.001. Statistical significance compared with respective control groups (all *P*-values were obtained by one-way ANOVA).

Mitochondria maintain their quality by regulating a balance between the processes of fission and fusion [12, 31]. We found that flubendazole increased DRP1 and p-DRP1^Ser616^ in MDA-MB-231 and MCF-7 cells, suggesting the proposition that the phosphorylation of Drp1 at the Ser616 site promotes its mitochondrial translocation, leading to mitochondrial fission (Fig. 3A). Similar results were obtained concerning the colocalization of DRP1 and mitochondria. (Fig. S2A, B). It has been reported that increased mitochondrial fission is required for mitophagy that eventually removes damaged mitochondria [32, 33]. Next, we tested whether mitochondrial fission played a critical role in flubendazole-induced mitophagy in breast cancer. Here we used *DRP1* shRNA to interfere with mitochondrial fission induced by flubendazole in MDA-MB-231 and MCF-7 cells. *DRP1* knockdown markedly attenuated flubendazole-induced colocalization of the autophagosome with the mitochondria (Fig. 3B–D). Similarly, western blot and immunofluorescence analysis revealed that *DRP1* shRNA decreased the expression of PINK1 and Parkin treated by flubendazole (Fig. 3E–G). Moreover, pretreatment with DRP1 inhibitor mdivi-1 attenuated flubendazole-induced DRP1, Parkin and PINK1 levels (Fig. S3). Altogether, these results implicate DRP1-mediated mitochondrial fission as an early upstream event of flubendazole-treated mitophagy.

However, excessive fission results in an increase in the fragmentation of mitochondria and loss of their polarization [33]. We then addressed whether flubendazole-induced mitophagy plays a role in degrading damaged mitochondria. We found that block mitophagy using *DRP1* shRNA reduced the fragmented mitochondria and restored mitochondria length in flubendazole-treated MDA-MB-231 and MCF-7 cells (Fig. S4A). Following the restoration of mitochondria morphology, mtDNA copy number was significantly increased in *DRP1*-knockdown cells compared with the control cells in response to flubendazole treatment (Fig. S4B). Consistently, the impairment of mitophagy with *DRP1* shRNA efficiently mitigated the flubendazole-induced ATP loss and mitochondrial reactive oxygen species production (Fig. S4C–E). Similar results were obtained concerning the expression levels of mitochondrial components such as SOD2, VDAC1, COX IV, and TOM20, which were also partially attenuated by mitophagy inhibition in flubendazole-treated MDA-MB-231 and MCF-7 cells (Fig. S4F). Collectively, these data indicate that flubendazole induces mitochondrial dysfunction by DRP1-mediated mitophagy in breast cancer.

### Flubendazole-induced mitophagy inhibits cell proliferation in breast cancer

**Fig. 4:**
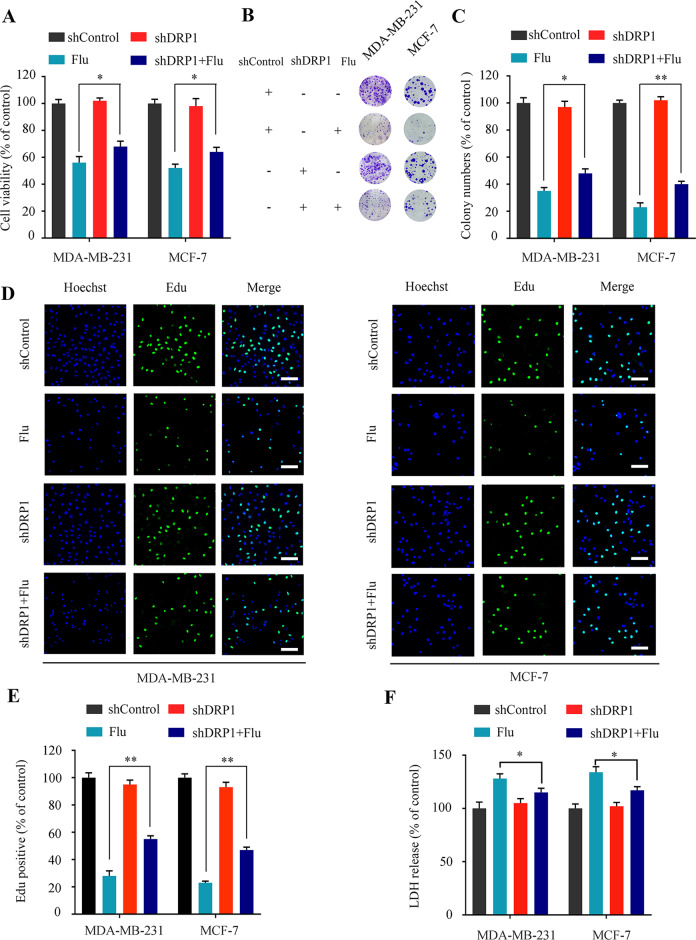
Flubendazole-induced mitophagy inhibits cell proliferation in flubendazole-treated MDA-MB-231 and MCF-7 cells. **A** MDA-MB-231 and MCF-7 cells were transfected with negative-control or *DRP1* shRNA for 24 h, respectively. After treatment with or without flubendazole (0.5 μM) for 24 h, cell viability was measured by MTT assay. **B**, **C** MDA-MB-231 and MCF-7 cells were transfected with negative-control or *DRP1* shRNA for 24 h, respectively. After treatment with or without flubendazole (0.5 μM) for two weeks. Representative images and quantification of colonies were shown. **D**–**F** MDA-MB-231 and MCF-7 cells were transfected with negative-control or *DRP1* shRNA for 24 h, respectively. After treatment with or without flubendazole (0.5 μM) for 24 h, then detected by Edu assay (**D**, **E**) and LDH release (**F**). Scale bar, 50 µm. Data represent mean ± SD. *P < 0.05, **P < 0.01, ***P < 0.001. Statistical significance compared with respective control groups (all *P*-values were obtained by one-way ANOVA).

Increasing evidence indicates that flubendazole induces autophagy and affects tumor cell proliferation [26, 34, 35]. We then addressed whether flubendazole-induced mitophagy inhibits cell proliferation in breast cancer. We observed that suppressing mitophagy by *DRP1*-knockdown could restore cell growth in flubendazole-treated MDA-MB-231 and MCF-7 cells (Fig. 4A–C). Additionally, compared to the control group, *DRP1*-knockdown increased the colocalization of Edu with Hoechst (Fig. 4D, E), suggesting that mitophagy contributes to proliferation inhibition in flubendazole-treated MDA-MB-231 and MCF-7 cells. We then performed LDH release assay and found that *DRP1*-knockdown alleviated flubendazole-induced cytotoxicity in MDA-MB-231 and MCF-7 cells (Fig. 4F). We also found that blocking mitophagy using *Parkin* shRNA increased cell viability and colony formation in flubendazole-treated MDA-MB-231 and MCF-7 cells (Fig. S5A–C). Consistently, the impairment of mitophagy with *Parkin* shRNA partially mitigated the flubendazole-induced proliferation inhibition (Fig. S5D–F). These results demonstrate that flubendazole-induced mitophagy inhibits cell proliferation in breast cancer.

### Flubendazole-induced mitophagy enhances anti-migration potential in MDA-MB-231 cells

**Fig. 5:**
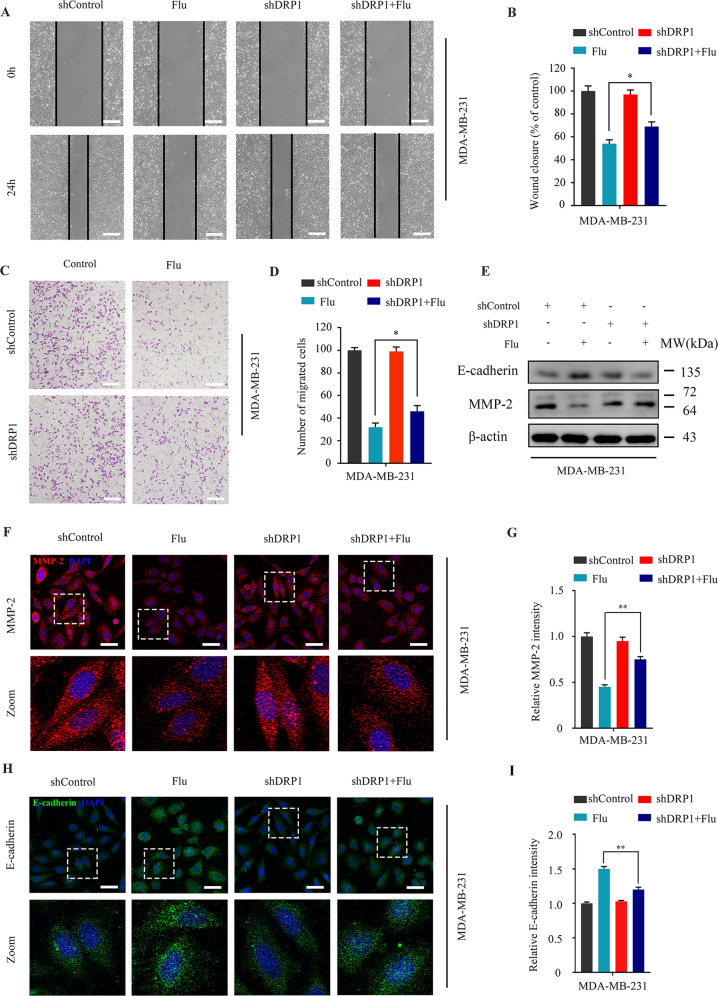
Flubendazole-induced mitophagy enhances anti-migration potential in MDA-MB-231 cells. MDA-MB-231 cells were transfected with negative-control or *DRP1* shRNA for 24 h and treatment with or without flubendazole (0.5 μM) for 24 h. **A**, **B** The scratch assay was used to measure the migration capabilities of the cells. Representative images and statistics were shown. Scale bar, 100 µm. **C**, **D** Transwell assay was used to measure the number of migrated cells. Representative images and statistics were shown Scale bar, 50 µm. **E** Immunoblotting of E-cadherin and MMP-2 expression. β-actin was measured as the loading control. **F**–**I** The expression of MMP-2 and E-cadherin were analyzed by immunofluorescence. Scale bar, 20 µm. Data represent mean ± SD. *P < 0.05, **P < 0.01, ***P < 0.001. Statistical significance compared with respective control groups (all *P*-values were obtained by one-way ANOVA).

Previous studies show that flubendazole could block tumor metastasis [24, 36, 37], and autophagy plays a catalytic role in this process [26]. Next, we sought to assess the association between migration and mitophagy. We found that *DRP1*-knockdown could increase the wound closure ratio and the number of migrating MDA-MB-231 cells (Fig. 5A–D). Western blot and immunofluorescence analysis showed that the ability of flubendazole to downregulate MMP-2 and up-regulate E-cadherin was also attenuated by *DRP1*-knockdown (Fig. 5E–I). Collectively, these data demonstrate that flubendazole-induced mitophagy enhances anti-migration potential in MDA-MB-231 cells.

### Flubendazole induces mitochondrial dysfunction and DRP1-mediated mitophagy in breast cancer via targeting EVA1A

**Fig. 6:**
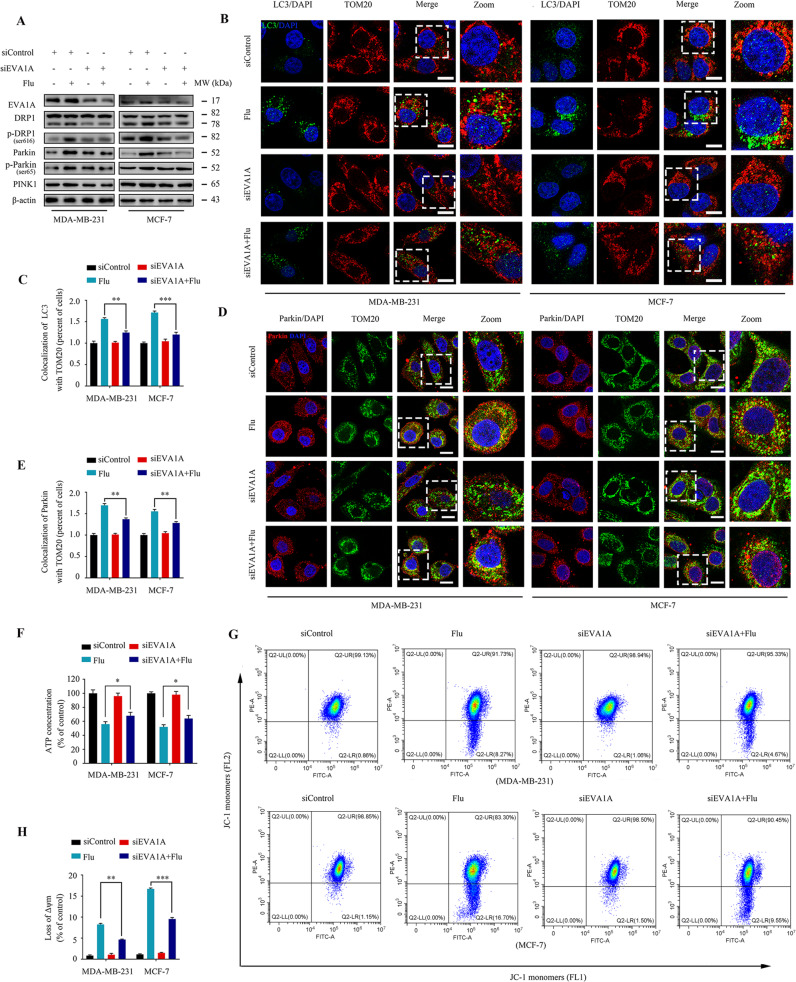
Flubendazole induces mitochondrial dysfunction and DRP1-mediated mitophagy via targeting EVA1A in MDA-MB-231 and MCF-7 cells. MDA-MB-231 and MCF-7 cells were transfected with *EVA1A* siRNA or negative-control for 24 h, respectively, followed by treatment with or without flubendazole (0.5 μM). **A** Immunoblotting of EVA1A, DRP1, p-DRP1^ser616^, Parkin, p-Parkin^ser65^ and PINK1 expression. β-actin was measured as the loading control. **B**, **C** The autophagosomes are labeled by LC3 (green fluorescence) protein, and the mitochondria are labeled by TOM20 (red fluorescence) protein. The number of co-localized LC3 and TOM20 was quantified. Scale bar, 10 µm. **D**, **E** Colocalization of Parkin (red fluorescence) protein and TOM20 (green fluorescence) protein in MDA-MB-231 and MCF-7 cells following flubendazole (0.5 μM, 24 h) treatment. The number of co-localized Parkin and TOM20 was quantified. Scale bar, 10 µm. **F** ATP content measurement. **G**, **H** Flow cytometric analysis and quantification of mitochondrial membrane potential changes. Data represent mean ± SD. *P < 0.05, **P < 0.01, ***P < 0.001. Statistical significance compared with respective control groups (all *P*-values were obtained by one-way ANOVA).

We have previously demonstrated that EVA1A plays an essential role in TNBC through autophagy and apoptosis-related mechanisms [26]. Hence, to explore whether flubendazole induced mitophagy by targeting EVA1A, the specific small-interfering RNA was transfected into MDA-MB-231 and MCF-7 cells to silence *EVA1A* expression. We found that *EVA1A* knockdown partially blocked the expression of DRP1 and phosphorylation at Ser616 in flubendazole-treated cells (Fig. 6A). We then observed that *EVA1A* siRNA markedly attenuated flubendazole-induced colocalization of the autophagosome with the mitochondria (Fig. 6B, C). We also verified that *EVA1A* gene silencing decreased PINK1, Parkin and p-Parkin^ser65^ expression in MDA-MB-231 and MCF-7 cells (Fig. 6A). Similar results were obtained concerning the immunofluorescence analysis of Parkin and TOM20 (Fig. 6D, E), which indicate that flubendazole induces DRP1-mediated mitophagy via targeting EVA1A in breast cancer. Moreover, we found that *EVA1A* gene silencing mitigated the flubendazole-induced ATP loss and decline of mitochondrial membrane potential in MDA-MB-231 and MCF-7 cells (Fig. 6F–H). Together, flubendazole induces mitochondrial dysfunction and DRP1-mediated mitophagy in breast cancer via targeting EVA1A.

### EVA1A overexpression induces DRP1-mediated mitophagy and exerts anti-cancer effects in breast cancer

**Fig. 7:**
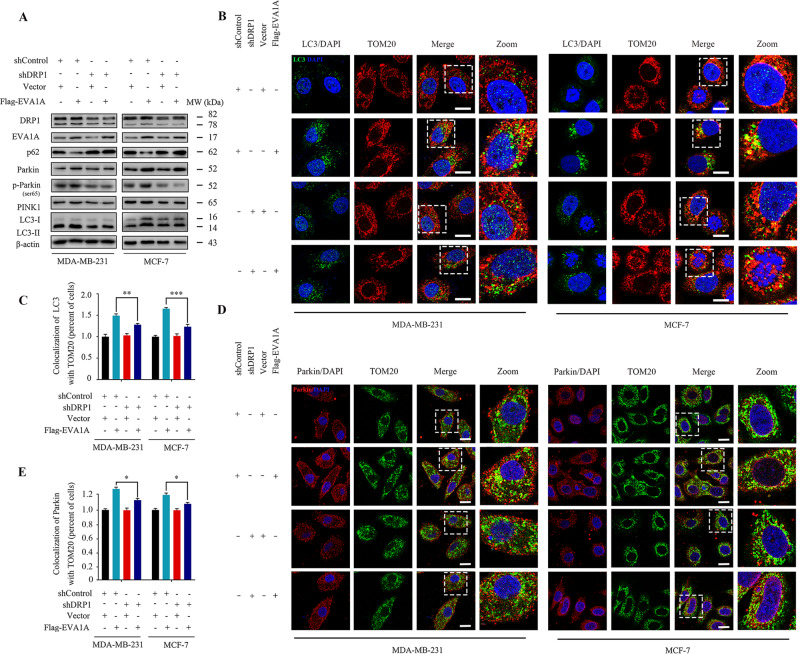
EVA1A overexpression induces DRP1-mediated mitophagy in MDA-MB-231 and MCF-7 cells. MDA-MB-231 and MCF-7 cells were co-transfected with *DRP1* shRNA and Flag-EVA1A or vehicle control respectively for 48 h. **A** Immunoblotting of DRP1, EVA1A, p62, Parkin, p-Parkin^ser65^, PINK1, and LC3 expression. β-actin was measured as the loading control. **B**, **C** The autophagosomes are labeled by LC3 (green fluorescence) protein, and the mitochondria are labeled by TOM20 (red fluorescence) protein. The number of co-localized LC3 and TOM20 was quantified. Scale bar, 10 µm. **D**, **E** Colocalization of Parkin (red fluorescence) protein and TOM20 (green fluorescence) protein in MDA-MB-231 and MCF-7 cells following flubendazole (0.5 μM, 24 h) treatment. The number of co-localized Parkin and TOM20 was quantified. Scale bar, 10 µm. Data represent mean ± SD. *P < 0.05, **P < 0.01, ***P < 0.001. Statistical significance compared with respective control groups (all *P*-values were obtained by one-way ANOVA).

To further understand the mechanism underlying between EVA1A and DRP1-mediated mitophagy. Then, we explored the effect of EVA1A overexpression on mitophagy in DRP1-depleted MDA-MB-231 and MCF-7 cells. We found that EVA1A overexpression could increase the formation of fluorescent autophagosomes (in yellow) and autolysosomes (in red). However, *DRP1*-knockdown reduced the accumulation of autophagosomes and autolysosomes (Fig. S6A, B). Likewise, we found that *DRP1*-knockdown partially suppressed the upregulation of Parkin, p-Parkin^ser65^, PINK1 and LC3 in EVA1A overexpression MDA-MB-231 and MCF-7 cells (Fig. 7A). We also observed that *DRP1*-knockdown decreased the colocalization of LC3 with TOM20 in EVA1A overexpression MDA-MB-231 and MCF-7 cells (Fig. 7B, C). Moreover, *DRP1*-depleted MDA-MB-231 and MCF-7 cells showed a decrease in the colocalization of Parkin with TOM20 induced by EVA1A overexpression (Fig. 7D, E). Therefore, these data indicate that EVA1A overexpression induces DRP1-mediated mitophagy.

**Fig. 8:**
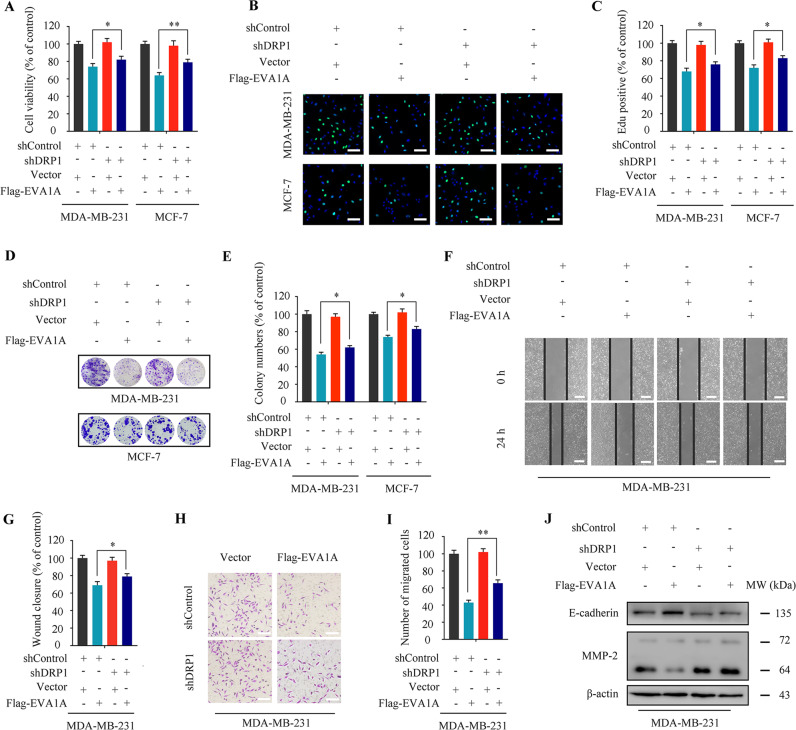
Silencing DRP1 partially blocks the anti-proliferative and anti-migration effects of EVA1A overexpression in breast cancer. MDA-MB-231 and MCF-7 cells were co-transfected with *DRP1* shRNA and Flag-EVA1A or vehicle control respectively for 48 h. **A** Cell viability was measured by MTT assay. **B**, **C** Representative images and quantification of Edu-positive cells were shown. Scale bar, 50 µm. **D**, **E** Representative images and quantification of colonies were shown. **F**, **G** The scratch assay was used to measure the migration capabilities of the cells. Representative images and statistics were shown. Scale bar, 100 µm. **H**, **I** Transwell assay was used to measure the number of migrated cells. Representative images and statistics were shown. Scale bar, 50 µm. **J** Immunoblotting of E-cadherin and MMP-2 expression. β-actin was measured as the loading control. Data represent mean ± SD. *P < 0.05, **P < 0.01, ***P < 0.001. Statistical significance compared with respective control groups (all *P*-values were obtained by one-way ANOVA).

Next, we analyzed the effect of EVA1A overexpression on cell proliferation and migration in normal and *DRP1*-depleted MDA-MB-231 and MCF-7 cells. We observed that EVA1A overexpression could significantly inhibit cell growth and proliferation in normal cells, but this inhibitory effect was attenuated in *DRP1*-depleted cells (Fig. 8A–E). Indeed, compared to the normal cells, *DRP1*-depleted cells showed a decline in cytotoxicity induced by EVA1A overexpression (Fig. S7A). After that, we investigated the effect of EVA1A overexpression on cell migration in *DRP1*-depleted MDA-MB-231 cells. Interestingly, we found that *DRP1*-depleted MDA-MB-231 cells had a higher migration capacity than EVA1A overexpressed wild-type cells (Fig. 8F–I). This observation was consistent with MMP-2 down-regulation and E-cadherin upregulation, as described by western blot and immunofluorescence in MDA-MB-231 cells (Fig. 8J and Fig. S7B–E). Collectively, EVA1A overexpression triggers excessive DRP1-mediated mitophagy and exerts anti-cancer effects in breast cancer.

## Discussion

In this study, we investigated the roles and mechanisms involved in mitophagy in response to the anti-cancer effects of flubendazole in breast cancer. Our data first suggested that flubendazole impairs mitochondrial outer membrane permeability and mitochondrial function, accompanied by mitophagy. We found that flubendazole increased DRP1 expression, resulting in PINK1 aggregation and parkin mitochondrial translocation, promoting excessive mitophagy. The resultant excessive mitophagy contributed to mitochondrial damage and dysfunction induced by flubendazole, thus inhibiting breast cancer cells proliferation and migration. Interestingly, we demonstrated that excessive DRP1-mediated mitophagy is characterized as a critical event in response to the anti-tumor effects of EVA1A in breast cancer. To our knowledge, our finding provides a novel mechanism of flubendazole against breast cancer with a focus on the mitochondrial dysfunction and DRP1-mediated mitophagy in breast cancer via targeting EVA1A.

Mitochondria are essential mediators of tumorigenesis, as this process requires flexibility to adapt to cellular and environmental alterations [38–40]. Owing to its vital role in cell proliferation and death, mitochondria have emerged as critical pharmacological targets [41–43]. Here, we initially demonstrated that flubendazole provokes increased mitochondrial outer membrane permeability and a decrease in mitochondrial membrane potential, accompanied by an accelerated release of Cyto C from mitochondria into the cytosol. Meanwhile, flubendazole treatment increased the number of mitochondria with ring-shaped structures compared with the normal tubular mitochondria. Moreover, the mitochondria, known as the “power source of the cell,” produced less ATP and more superoxide after flubendazole treatment, which indicates that flubendazole impairs mitochondrial function. In addition, flubendazole induced mitochondrial fission by promoting the phosphorylation of Drp1 at Ser616.

In line with autophagy, mitophagy plays a double-faceted role in tumorigenesis in response to various stress conditions [44, 45]. Generally, mitophagy degrades the damaged mitochondria and prompts tumor cells to rapidly adapt to these hostile conditions, thereby supporting cell proliferation and evading activation of cell death programs [46]. However, excessive mitophagy impairs the stability of the mitochondrial microenvironment and contributes to tumor cells death [14]. For example, polyphyllin I induce mitophagy and apoptotic cell death in breast cancer [47]. Furthermore, in other carcinomas such as HCC cells, ketoconazole exacerbates mitophagy to induce apoptosis, and melatonin is sensitive to the cytotoxic effect of sorafenib via mitophagy induction [48, 49]. Consistent with these findings, our results show that flubendazole promotes mitophagy via PINK1/Parkin signaling, and blocking mitophagy using Parkin *shRNA* increased proliferation of breast cancer cells. Moreover, inhibition of DRP1 prevents PINK1 accumulation and Parkin recruitment, accompanied by alleviation of mitochondrial dysfunction and anti-cancer efficiency in response to flubendazole treatment, suggesting that DRP1-mediated excessive mitophagy may lead to cell death. These results indicate that excessive or lethal mitophagy may be a potential target for breast cancer therapy.

EVA1A, an endoplasmic reticulum-associated protein, interacts with ATG16L1 and promotes ATG12-ATG5/ATG16L1 complex recruitment to the autophagic membrane and enhances the autophagosome formation [50]. Moreover, EVA1A induces cell death in many tumors via regulating autophagic and apoptotic mechanisms [51–56]. We previously reported that flubendazole could restore the expression of EVA1A in TNBC cells, therefore inducing autophagic cell death and eliciting anti-cancer effects [26]. In this regard, our results presented herein demonstrate for the first time that EVA1A overexpression induced mitophagy in breast cancer. Likewise, flubendazole induces mitochondrial dysfunction and DRP1-mediated mitophagy in breast cancer via targeting EVA1A. Interestingly, our results support that DRP1-mediated mitophagy is involved in the anti-cancer effects of EVA1A, as evidenced by silencing DRP1 blocks the anti-proliferative and anti-migration effects of EVA1A overexpression in breast cancer. Moreover, considering EVA1A acts as an adaptor protein to recruit or bind proteins in the lysosome or endoplasmic reticulum [57], we believe that with the deepening of the research, there may be other mechanisms related to the anti-cancer effects of EVA1A and deserve better clarification.

In summary, our results suggest that DRP1-mediated mitophagy induced by EVA1A overexpression may be the primary contributing factor for mitochondrial damage and dysfunction in breast cancer cells in response to flubendazole treatment, resulting in inhibition of cell proliferation and migration. These findings would provide new insights into the molecular mechanisms in relation to the anti-tumor activities of flubendazole, and may be conducive to its rational use in potential clinical applications.

## Materials and methods

### Cell culture and reagents

The human breast cancer cell lines MDA-MB-231 and MCF-7 were purchased from American Type Culture Collection (ATCC, Manassas, VA, USA). The cells were maintained in Dulbecco’s modified Eagle medium (DMEM) medium containing 10% fetal bovine serum (FBS, Gibco; Thermo Fisher Scientific, USA) and 1% penicillin-streptomycin (Life Technologies, Grand Island, NY, USA) in 5% CO_2_ at 37 °C. Cells were grown to 70-80% confluence in cell culture dishes or plates, and all the experiments were performed on logarithmically growing cells.

Flubendazole (SML2510), Mdivi-1 (475856), MTT (M2128), DAPI (D9542) were purchased from Sigma-Aldrich (St. Louis, MO, USA). Antibodies used in this study were as follow: DRP1 (1:1000, 184247, Abcam, USA), p-DRP1^Ser616^ (1:1000, 3455, CST, USA), Mitofusin-2 (1:1000, 9482, CST, USA), VDAC1 (1:1000, 55259, Proteintech, USA), SOD2 (1:1000, 13194, CST, USA), COX IV (1:1000, 4844, CST, USA), TOM20 (1:1000, 42406, CST, USA), PINK1 (1:500, 23274-1-AP, Proteintech), Parkin (1:500, 14060-1-AP, Proteintech, USA), p-Parkin^ser65^ (1:1000, 36866, CST, USA), LC3 (1:1000, 51520, Abcam, USA), p62 (1:1000, 8025, CST, USA), MMP-2 (1:1000, 87809, CST, USA), E-cadherin (1:1000, 14472, CST, USA), EVA1A (1:1000, 216043, Abcam, USA), β-actin (1:1000, 66009-1-Ig, Proteintech, USA), MitoTracker^TM^ Deep Red FM (M22426, Invitrogen, USA).

### Cell viability assay

MDA-MB-231 and MCF-7 cells were dispensed in 96-well plates (6 × 10^3^). After incubation at 37 °C for 24 h, cells were treated with different concentrations of flubendazole for the indicated time periods. Cell viability was measured by MTT assay.

### Edu cell-proliferation assay

MDA-MB-231 and MCF-7 cells (3 × 10^4^) after different transfections were seeded in 12-well plates and maintained for 24 h. Then, cells were further incubated with 10 μM Edu reagent for 2 h and then performed according to the manufacture’s instruction (Beyotime; Cat: C0071S, China). The number of Edu-stained cells was analyzed under a fluorescence microscope.

### LDH release assay

MDA-MB-231 and MCF-7 cells after different transfections were seeded in 96-well plates and maintained for 24 h. Then, LDH release assays were performed according to the manufacturer’s instructions (Beyotime, Cat: C0016, China).

### ATP measurement

MDA-MB-231 and MCF-7 cells after different transfections were seeded in 96-well plates and maintained for 24 h. Then, ATP measurements were performed according to the manufacturer’s instructions (Beyotime; Cat: S0026, China).

### Colony formation assay

A colony formation assay was performed as previously described [58]. Briefly, the MDA-MB-231 and MCF-7 cells were cultured in 6-well plates and treated with the indicated concentration of flubendazole or vehicle control for 14 days. Then, cells were fixed with 4% paraformaldehyde and stained with crystal violet (Beyotime; Cat: C0121, China). The number of colonies was counted.

### GFP/mRFP-LC3 transfection

The MDA-MB-231 and MCF-7 cells (2.5 × 10^4^ cells/well) were cultured in 24-well culture plates. After incubation of 24 h, cells were transfected with GFP/mRFP-LC3 (HB-AP2100001, HANBIO, China) for 6 h. Then the cells were used for subsequent experiments 36 h later and were observed under a fluorescence microscope.

### Mitochondrial permeability transition pore (mPTP) opening

The opening of the mPTP was measured using an mPTP assay kit and analyzed with a fluorescence microscope. The detailed procedures were performed according to the corresponding manufacturer’s instructions (Beyotime; C2009S, China).

### Mitochondrial membrane potential measurement

JC-1 staining was used to assess the mitochondrial membrane potential. The MDA-MB-231 and MCF-7 cells were incubated with mitochondrial fluorescent JC-1 prober and measured by flow cytometry (Becton Dickinson, Franklin Lakes, NJ, China). The detailed procedures were performed according to the corresponding manufacturer’s instructions (KGA603, KeyGEN BioTECH, China).

### Mitochondrial DNA quantification

Mitochondrial DNA was isolated with a Mitochondria DNA Isolation kit (Abcam, ab65321) followed by PCR analysis using the mtDNA primers. The mtDNA primers were designed to detect MT-CO2 (mitochondrially encoded cytochrome c oxidase II).

### Mitochondrial superoxide measurement

The MitoSOX^TM^ Red mitochondrial indicator was introduced to label the superoxide in the mitochondria (M36008, Invitrogen, USA). The MDA-MB-231 and MCF-7 cells were incubated with MitoSOX^TM^ Red FM according to the manufacturer’s instructions and subjected to flow cytometric analysis.

### Immunofluorescence analysis

Immunofluorescence assays were carried out according to a previous study [59]. The MDA-MB-231 and MCF-7 cells were cultured in 24-well plates. After treatment, cells were fixed with 4% paraformaldehyde in PBS for 30 min. The slides were then washed three times with PBS and incubated with 0.2% Triton X-100 (Sigma-Aldrich, 9002-93-1, USA) and 5% goat serum (Sigma-Aldrich, G9023, USA) for 30 min. Cells were incubated with indicated primary antibody overnight at 4 °C and subsequently incubated with secondary antibody (TRITC, ab6718; FITC, ab6717, Abcam, USA) at room temperature for 1 h. Nuclei were finally stained with DAPI for 5 min. Images were captured using a confocal laser scanning microscopy (Zeiss, Germany).

### Immunoblotting analysis

Immunoblotting assays were performed as previously described [60]. In brief, all cells were collected and lysed by lysis buffer at 4 °C for 30 min. The mitochondrial fraction was isolated using the Cell Mitochondria Isolation Kit (KGA827, KeyGEN BioTECH, China) based on the manufacturer’s instructions. The protein level of the supernatant was quantified by BCA protein assay (Biosharp, BL521A, China). Equal amounts of the total protein or mitochondrial protein were separated by 12% or 8% SDS-PAGE and electrophoretically transferred to 0.22 μm PVDF membranes (Millipore, USA). Subsequently, membranes were blocked with 5% nonfat dried milk. Proteins were detected using primary antibodies, followed by HRP-conjugated secondary antibodies, and visualized by employing ECL as the HRP substrate.

### Scratch assay

Scratch assay was carried out as previously described [26]. The MDA-MB-231 cells were cultured in 6-well plates and scratch-wounded by sterilized pipettes. Then the cells were washed with PBS and cultured with standard medium or flubendazole. After 24 h incubation, pictures were taken by a phase-contrast microscope.

### Transwell migration assay

Transwell migration assay was performed according to the previous study [26]. The MDA-MB-231 cells were resuspended in 24-well culture plates with flubendazole and seeded on transwell filters (8 μm pore size, Millipore, USA). Inoculate serum-free DMEM medium in the top chamber, and add DMEM supplemented with 10% FBS in the bottom chamber. After 12 h, cells on the top side of the filters were wiped by cotton swaps. Cells on the lower side were then fixed in 4% paraformaldehyde and stained with 0.1% crystal violet. Images were taken under an inverted microscope.

### Transfection

siEVA1A, siControl, shDRP1, shParkin, vector, Flag-EVA1A, were synthesized by Genechem (Shanghai, China). The sequences of the RNA or cDNA involved in this study are listed in Supplementary information. The RNA was transfected with Lipofectamine 3000 reagent (Thermo Fisher Scientific, USA) for 48 h according to the manufacturer’s protocol.

### Quantitative real-time PCR

Total RNA was extracted using Trizol reagent (Invitrogen, USA) according to the manufacturer’s instructions. Reverse transcription was performed using HiScript III-RT SuperMix Kit (R323-01, Vazyme, China). Real-time PCR reactions were performed using iQ SYBR Green Supermix (BIO-RAD, 1708882, China). Primers used for real-time PCR are listed in Supplementary Information.

### Transmission electron microscopy

TEM was performed for the confirmation and monitoring of autophagosomes and mitochondria. The samples were fixed with 3% glutaraldehyde in 0.1 M phosphate buffer at 4 °C for 2 h. After fixation and dehydration (Epon812), ultrathin sections (50 nm) were obtained using an ultramicrotome (EM UC7). All the sections were stained with lead citrate (15 min) and uranyl acetate (2 min) and detected under a transmission electron microscope (JEM-1400 FLASH, JEOL, Japan).

### Statistical analysis

At least three independent experiments confirmed all the presented data and results. The data were expressed as means ± SEM and analyzed with GraphPad Prism 7.0 software. Statistical differences between two groups were determined using Student’s *t*-test, while multiple groups were determined using one-way analysis of variance. *P* < 0.05 was considered statistically significant.

## Supplementary information

Supplemental Materials

Original Data File

checklist

Author change agreement

## Data Availability

All data needed to evaluate the conclusions in the paper are present in the paper. Additional data related to this paper may be requested from the corresponding author.
