# Supplementary material for: Flubendazole induces mitochondrial dysfunction and DRP1-mediated mitophagy by targeting EVA1A in breast cancer
Source: Cell Death Dis. 2022 Apr 19;13(4):375. doi: 10.1038/s41419-022-04823-8 (PMC9019038; doi:10.1038/s41419-022-04823-8)
Supplement: Supplementary file 1 — Supplemental Materials [file 41419_2022_4823_MOESM1_ESM.docx]

**Supplemental Materials**

**Flubendazole induces mitochondrial dysfunction and DRP1-mediated mitophagy by targeting EVA1A in breast cancer**

Yongqi Zhen^1,2^, Zhaoxin Yuan^1^, Jiahui Zhang^1^, Yao Chen^1^, Yuning Fu^1^, Yi Liu^1^, Leilei Fu^1,*^, Lan Zhang^1,^*, Xian-Li Zhou^1,2,^*

^1^ Sichuan Engineering Research Center for Biomimetic Synthesis of Natural Drugs, School of Life Science and Engineering, Southwest Jiaotong University, Chengdu 610031, China

^2^ Key Laboratory of Advanced Technologies of Materials, Ministry of Education, Southwest Jiaotong University, 610031, Chengdu, China

*****Corresponding authors: Leilei Fu, E-mail: leilei_fu@163.com; Lan Zhang, E-mail: zhanglanx_9@126.com; Xian-Li Zhou, E-mail: zhouxl@swjtu.edu.cn.

1. **Supplementary Figures**

**Supplementary Figure. S1**

**
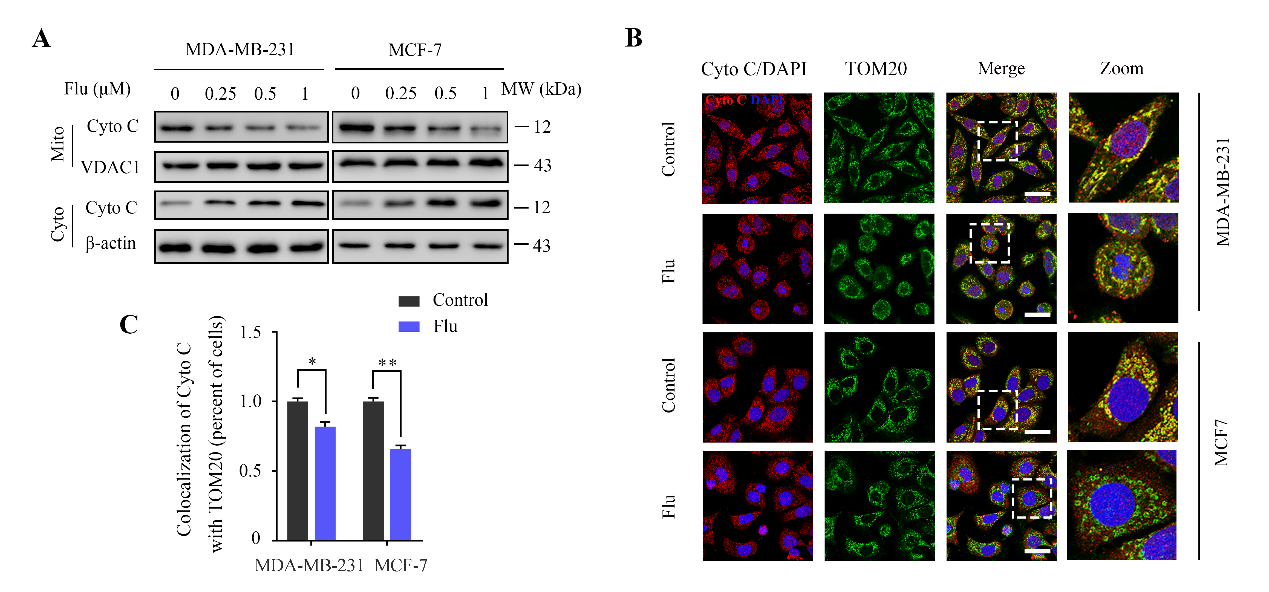
**

**Fig. S1 Flubendazole impairs the permeability of the mitochondrial outer membrane in MDA-MB-231 and MCF-7 cells. A** Immunoblotting analysis of Cyto C expression in MDA-MB-231 and MCF-7 cells treated with the indicated concentration of flubendazole for 24 h. β-actin (cytoplasmic fraction) and VDAC1 (mitochondrial fraction) were used as the loading controls. **B-C** Colocalization of Cyto C (red fluorescence) protein and TOM20 (green fluorescence) protein in MDA-MB-231 and MCF-7 cells following flubendazole (0.5 μM, 24 h) treatment. The number of co-localized Cyto C and TOM20 was quantified. Scale bar, 20 µm. Data represent mean ± SD. *, *P <* 0.05, **, *P <* 0.01. Statistical significance compared with respective control groups (all *p*-values were obtained by one-way ANOVA).

**Supplementary Figure S2**

**
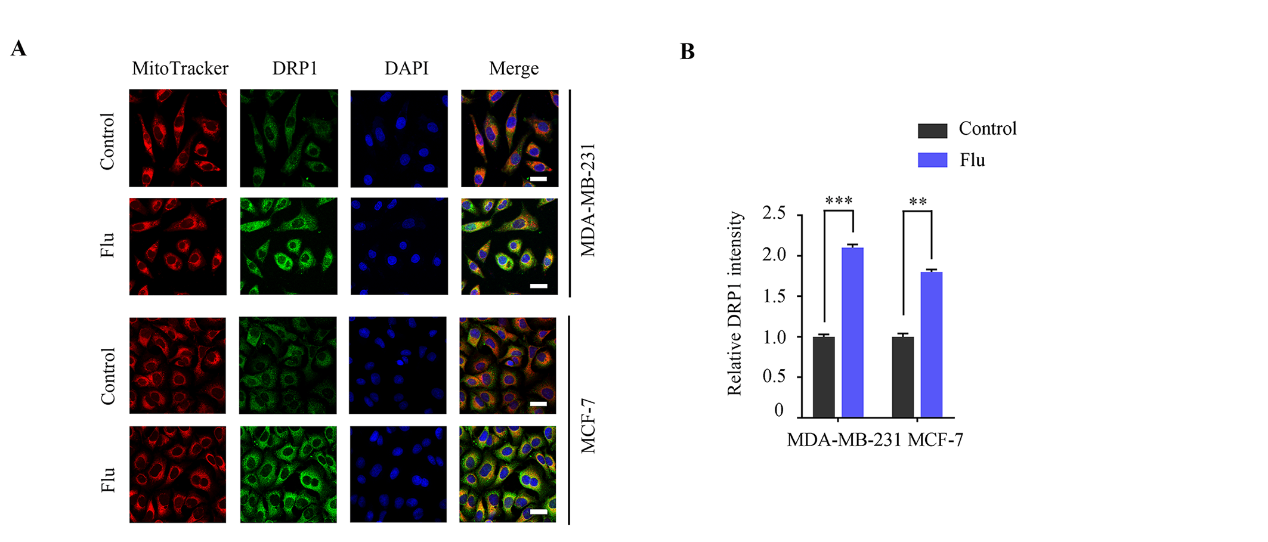
**

**Fig. S2 Flubendazole increased the expression of DRP1 in MDA-MB-231 and MCF-7 cells.** **A-B** MDA-MB-231 and MCF-7 cells were treated with or without flubendazole (0.5 μM) for 24 h and stained with MitoTracker^TM^ Deep Red FM probes for 30 min. After immunostaining with DRP1 and observed with a confocal microscope. The number of co-localized DRP1 and MitoTracker^TM^ Deep Red was quantified. Scale bar, 20 µm. Data represent mean ± SD. **, *P <* 0.01, ***, *P <* 0.001. Statistical significance compared with respective control groups (all *p*-values were obtained by one-way ANOVA).

**Supplementary Figure S3**

**
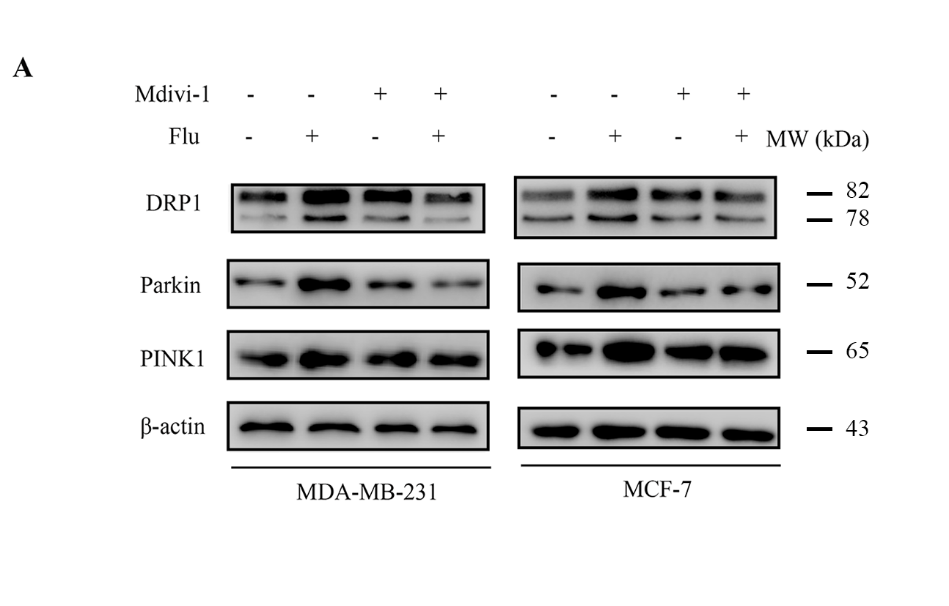
**

**Fig. S3 Inhibition of mitochondrial fission prevented flubendazole-induced mitophagy in MDA-MB-231 and MCF-7 cells. A** MDA-MB-231 and MCF-7 cells were pretreated with or without mdivi-1 (10 μM) for 2 h, followed by treatment with flubendazole (0.5 μM) for 24 h, then the expression levels of DRP1, Parkin and PINK1 were detected. β-actin was measured as loading control.

**Supplementary Figure S4**

**
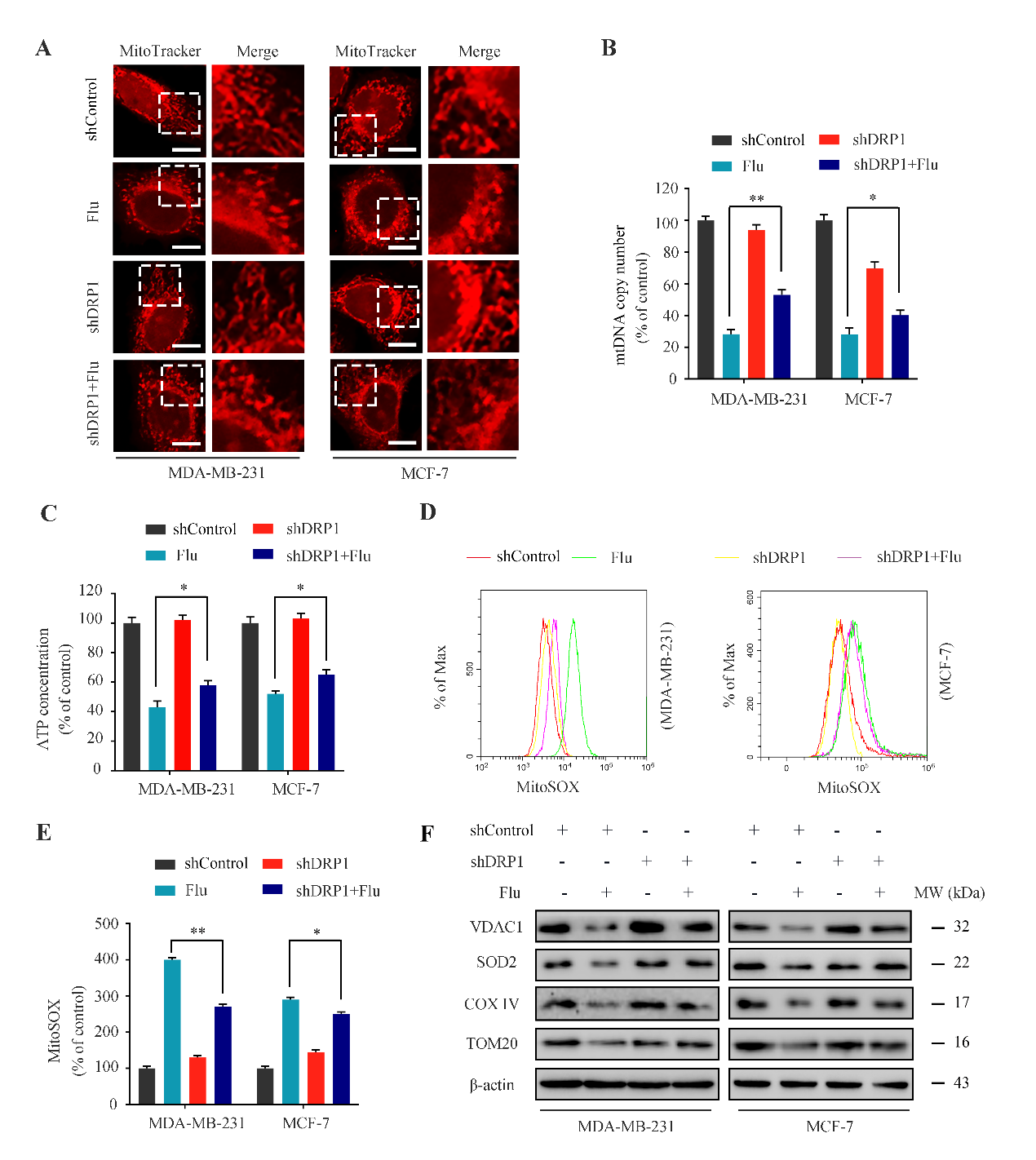
**

**Fig. S4 Flubendazole induces mitochondrial dysfunction by DRP1-mediated mitophagy in MDA-MB-231 and MCF-7 cells.** MDA-MB-231 and MCF-7 cells were transfected with negative-control or *DRP1* shRNA for 24 h, respectively. After treatment with or without flubendazole (0.5 μM) for 24 h and stained with MitoTracker^TM^ Deep Red FM probes for 30 min. **A** Mitochondrial morphology was observed by a confocal microscope. Representative images of mitochondrial morphology were shown, Scale bar, 5 µm. **B** RT-qPCR analysis of mitochondrial DNA copies in MDA-MB-231 and MCF-7 cells. **C** MDA-MB-231 and MCF-7 cells were transfected with negative-control or *DRP1* shRNA for 24 h, respectively. After treatment with or without flubendazole (0.5 μM) for 24 h and detected ATP content. **D-E** MDA-MB-231 and MCF-7 cells were transfected with negative-control or *DRP1* shRNA for 24 h, respectively. After treatment with or without flubendazole (0.5 μM) for 24 h and flow cytometric analysis of mitochondrial ROS accumulation. Representative images and quantification of mitochondrial ROS were shown. **F** MDA-MB-231 and MCF-7 cells were transfected with negative-control or *DRP1* shRNA for 24 h, respectively. After treatment with or without flubendazole (0.5 μM) for 24 h and immunoblotting analysis of VDAC1, SOD2, COX IV, TOM20 expression. β-actin was used as the loading control. Data represent mean ± SD. *, *P* < 0.05, **, *P <* 0.01. Statistical significance compared with respective control groups (all *p*-values were obtained by one-way ANOVA).

**Supplementary Figure S5**

**
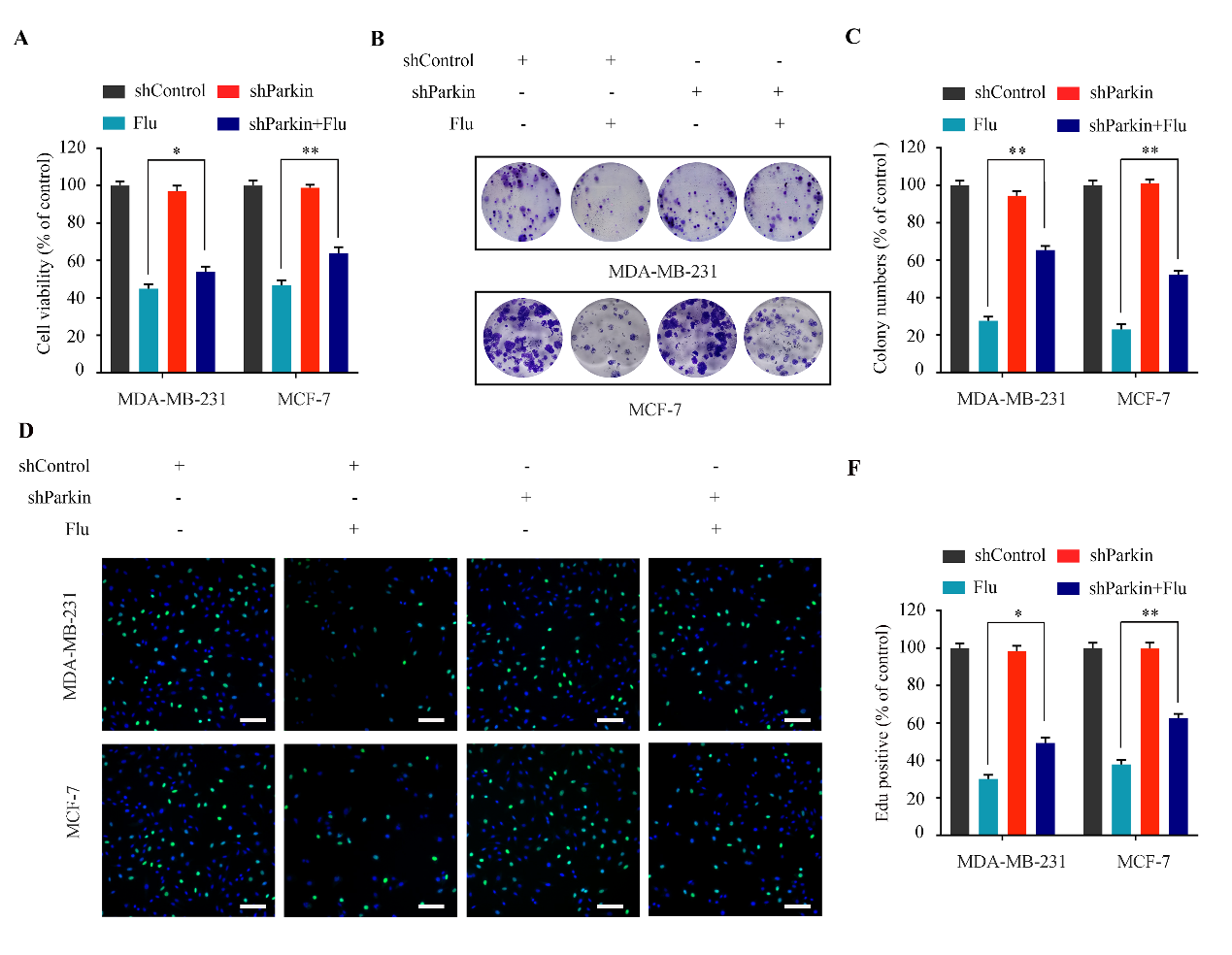
**

**Fig. S5 DRP1-mediated mitophagy potentiates proliferation inhibition in flubendazole-treated MDA-MB-231 and MCF-7 cells. A** MDA-MB-231 and MCF-7 cells were transfected with negative-control or *Parkin* shRNA for 24 h, respectively. After treatment with or without flubendazole (0.5 μM) for 24 h, cell viability was measured by MTT assay. **B-C** MDA-MB-231 and MCF-7 cells were transfected with negative-control or *Parkin* shRNA for 24 h, respectively. After treatment with or without flubendazole (0.5 μM) for two weeks. Representative images and quantification of colonies were shown. **D-F** Representative images and quantification of Edu-positive cells were shown. Scale bar, 50 µm. Data represent mean ± SD. *, *P <* 0.05, **, *P <* 0.01. Statistical significance compared with respective control groups (all *p*-values were obtained by one-way ANOVA).

**Supplementary Figure S6**

**
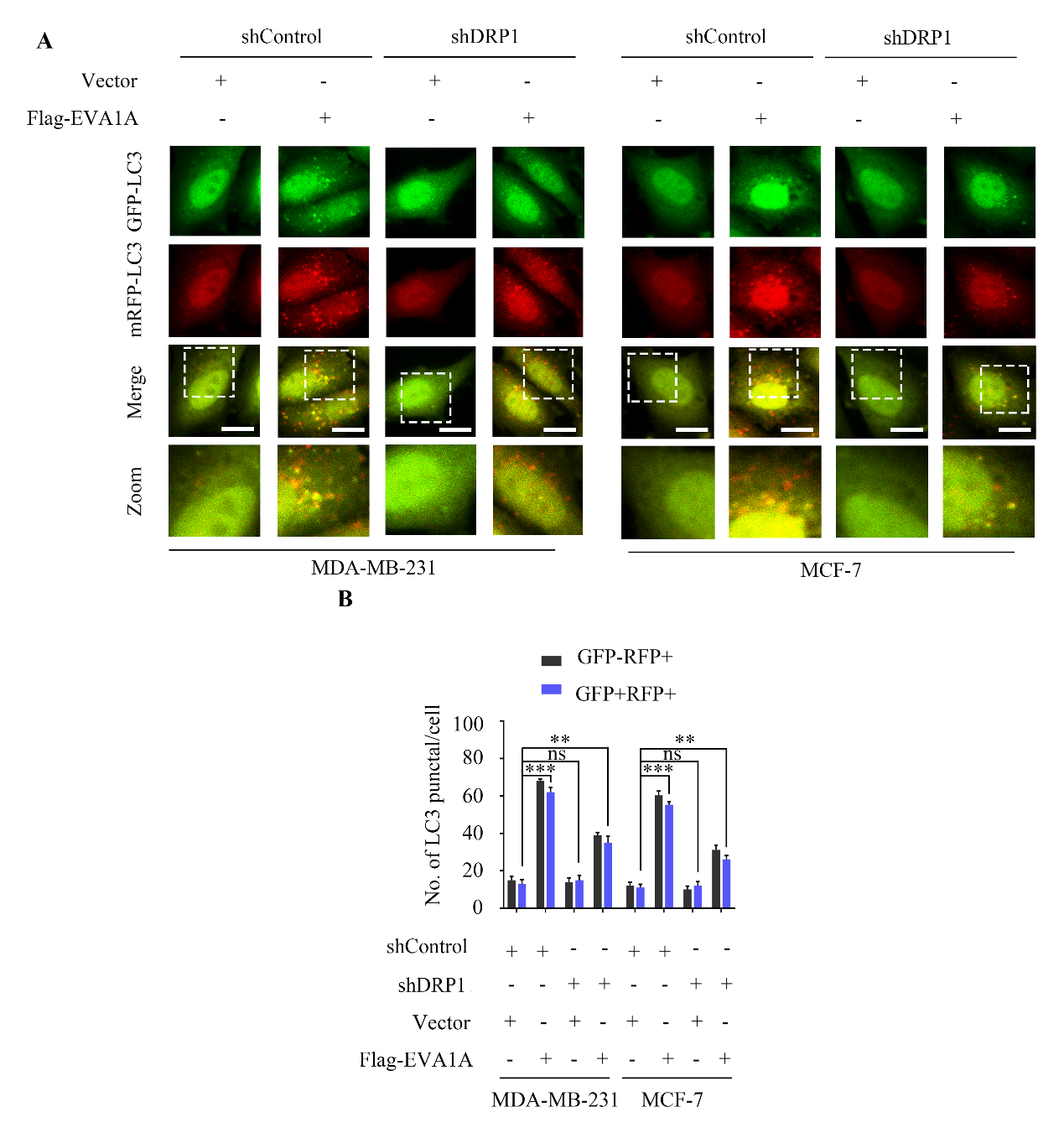
**

**Fig. S6 EVA1A overexpression induces DRP1-mediated mitophagy in MDA-MB-231 and MCF-7 cells. A-B** MDA-MB-231 and MCF-7 cells were transfected with GFP/mRFP-LC3 plasmid, after co-incubation with *DRP1* shRNA and Flag-EVA1A or vehicle control respectively for 48 h. Representative images and quantitative analysis of LC3 puncta were shown. Scale bar, 10 µm. Data represent mean ± SD. **, *P <* 0.01, ***, *P <* 0.001. Statistical significance compared with respective control groups (all *p*-values were obtained by one-way ANOVA).

**Supplementary Figure S7**


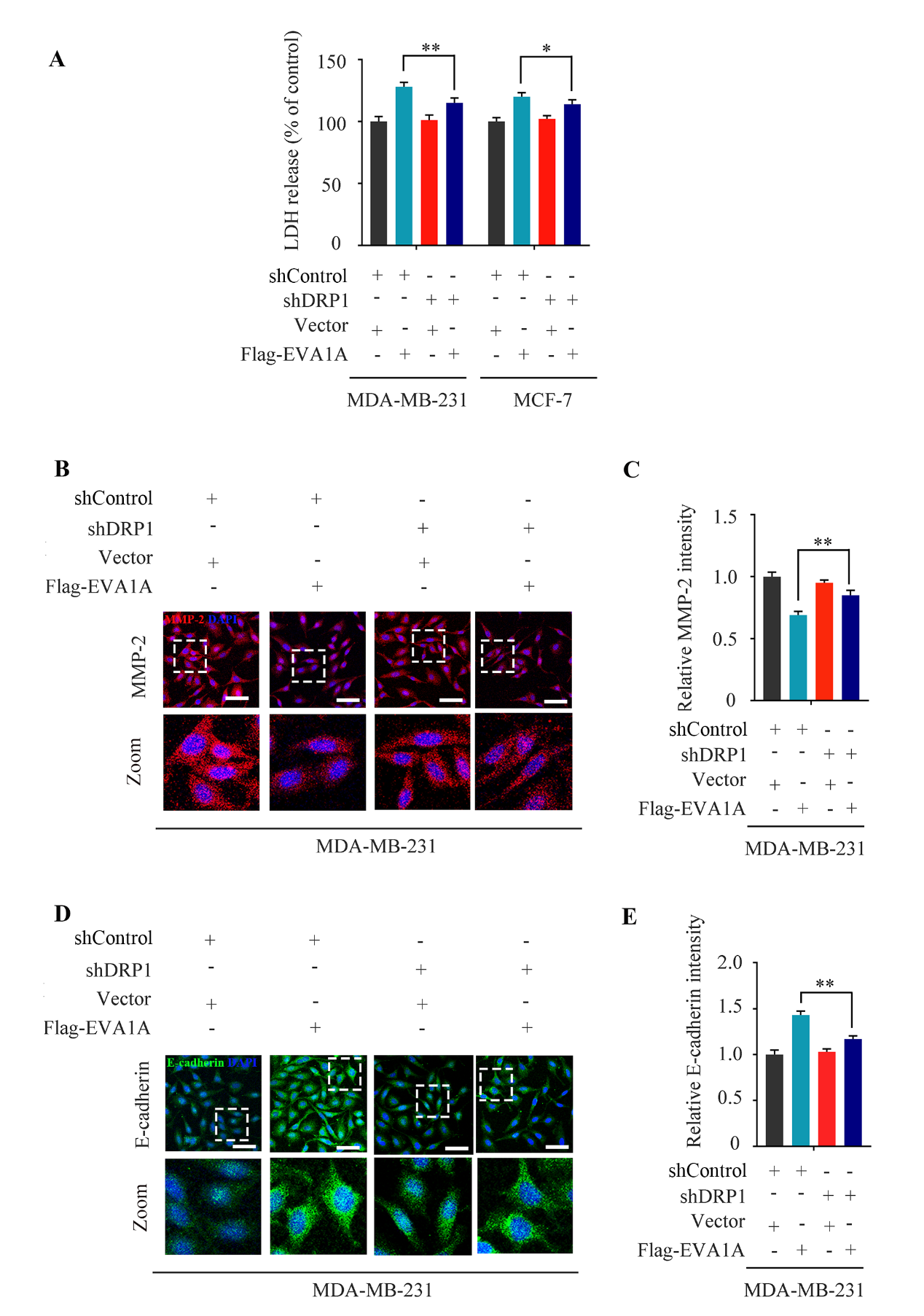


**Fig. S7 Silencing DRP1 partially blocks the anti-proliferative and anti-migration effects of EVA1A overexpression in breast cancer. A** MDA-MB-231 and MCF-7 cells were co-transfected with *DRP1* shRNA and Flag-EVA1A or vehicle control respectively for 48 h, then detected by LDH release. **B-E** MDA-MB-231 cells were co-transfected with *DRP1* shRNA and Flag-EVA1A or vehicle control respectively for 48 h. The expression of E-cadherin and MMP-2 were analyzed by immunofluorescence, Scale bar, 20 μm. Data represent mean ± SD. *P* < 0.05, **, *P <* 0.01. Statistical significance compared with respective control groups (all *p*-values were obtained by one-way ANOVA).**2. List of sequences-based reagents**

| **Primer name** | **Primer sequences (5’-3’)** |
| --- | --- |
| Human GAPDH forward | TGACAACAGCCTCAAGAT |
| Human GAPDH reverse | GAGTCCTTCCACGATACC |
| Human DRP1 forward | GCTGCTTCTGCTGAGGCTGATG |
| Human DRP1 reverse | TTGTGGACTGGCTGGCATAATTGG |
| Human MT-CO2 forward | CAAACCTACGCCAAAATCCA |
| Human MT-CO2 reverse | GAAATGAATGAGCCTACAGA |

| **Gene name** | **siRNA sense sequences (5’-3’)** |
| --- | --- |
| siEVA1A | GAGCCTGAATCGCTACTATTA |
| shDRP1 | GCTACTTTACTCCAACTTATT |
| shParkin | CGTGATTTGCTTAGACTGTTT |
| shControl | TTCTCCGAACGTGTCACGT |

| **Recombinant DNA** | **DNA sense sequences** |
| --- | --- |
| C-terminal Flag-tagged EVA1A | ATGAGGCTGCCCCTCAGCCACAGCCCAGAGCACGTGGAGATGGCTTTGCTCAGCAACATCCTAGCGGCCTATTCCTTTGTCTCAGAAAATCCTGAGCGAGCAGCTCTGTACTTTGTTTCTGGCGTGTGCATCGGGCTGGTGCTGACCCTGGCTGCTCTGGTGATAAGGATCTCTTGCCACACAGACTGCAGGCGGCGTCCCGGGAAGAAGTTCCTGCAGGACAGAGAGAGCAGCAGCGACAGCAGCGACAGCGAGGATGGCAGTGAGGACACCGTGTCCGATCTCTCCGTGCGGAGACACCGCCGCTTCGAGAGGACTTTGAACAAGAATGTGTTCACCTCTGCGGAGGAGCTGGAGCGCGCCCAGCGGCTGGAGGAGCGCGAGCGCATCATCAGGGAGATCTGGATGAATGGCCAGCCTGAGGTGCCCGGGACCAGGAGCCTGAATCGCTACTATTAGGACTACAAGGATGACGATGACAAGGATTACAAAGACGACGATAAGGACTATAAGGATGATGACGACAAA |
