# Supplementary material for: Flubendazole induces mitochondrial dysfunction and DRP1-mediated mitophagy by targeting EVA1A in breast cancer
Source: Cell Death Dis. 2022 Apr 19;13(4):375. doi: 10.1038/s41419-022-04823-8 (PMC9019038; doi:10.1038/s41419-022-04823-8)

**Figure. 1J**


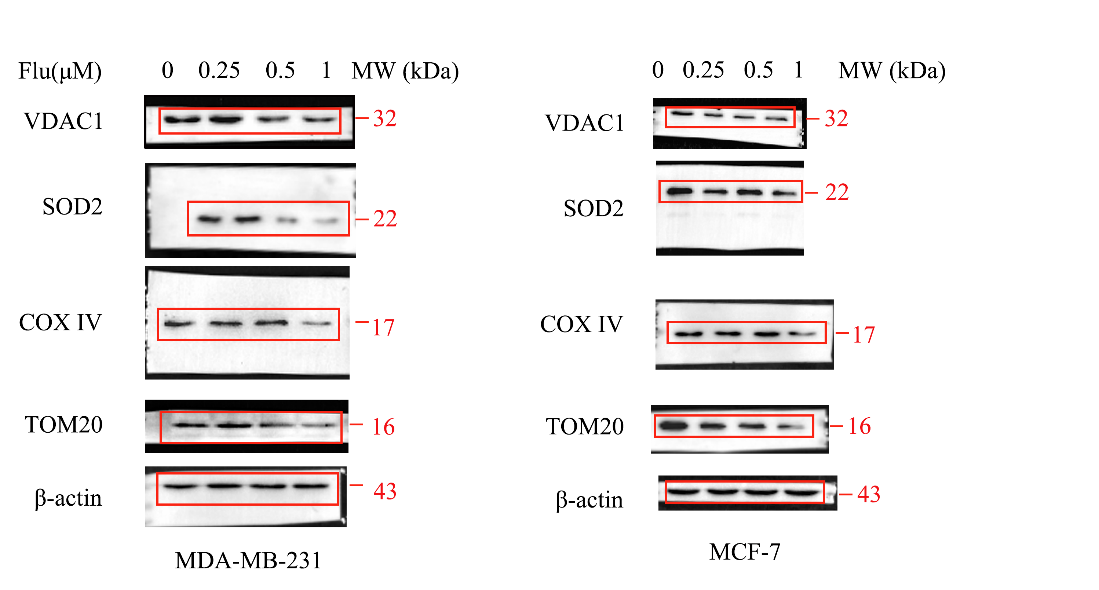


**Figure. 2D**


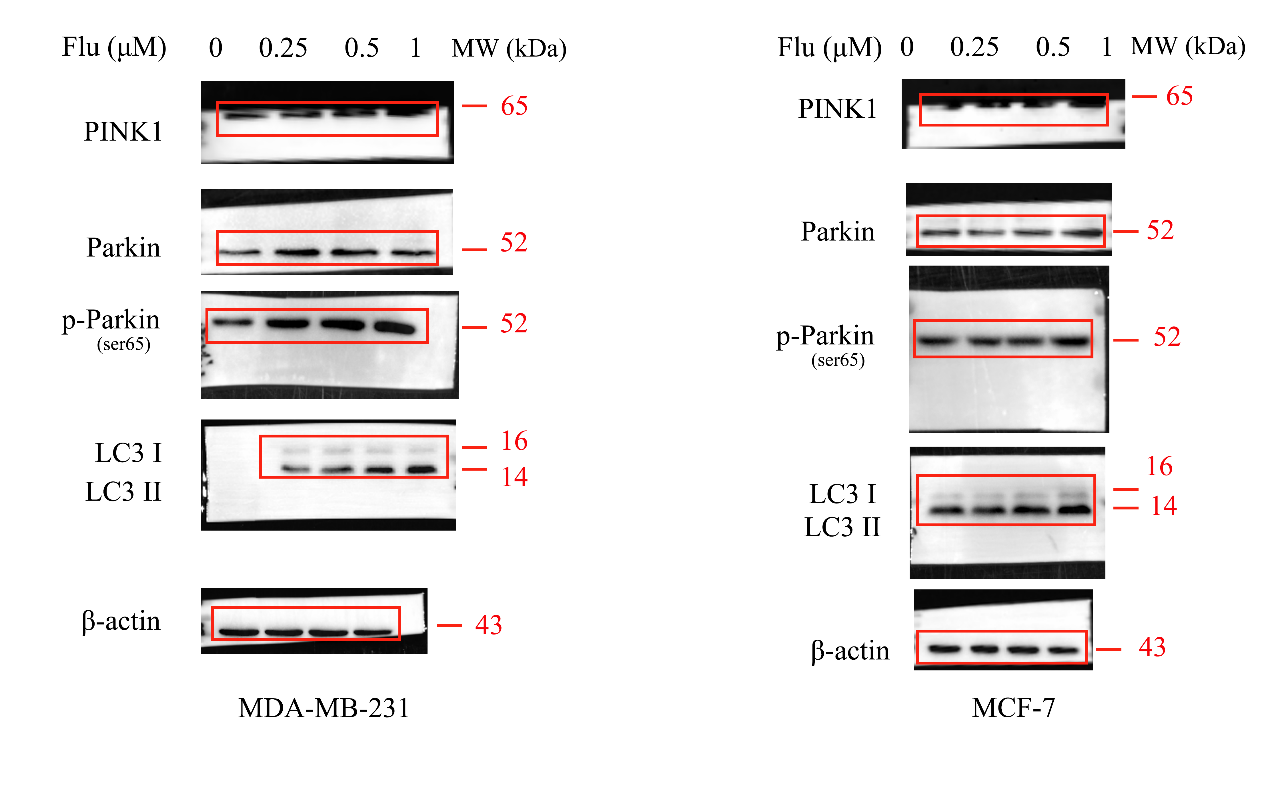


**Figure. 2E**


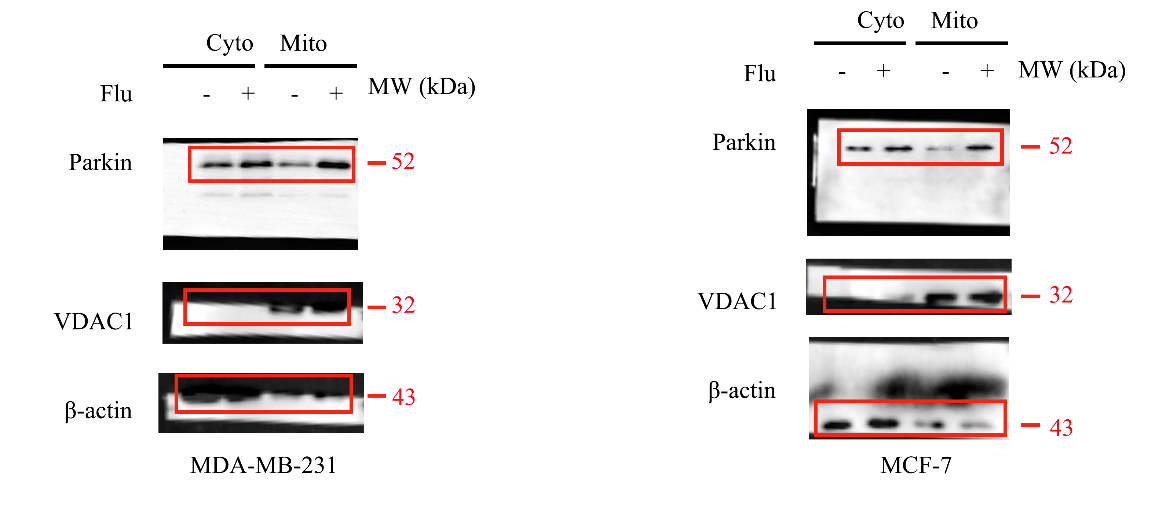


**Figure. 3A**


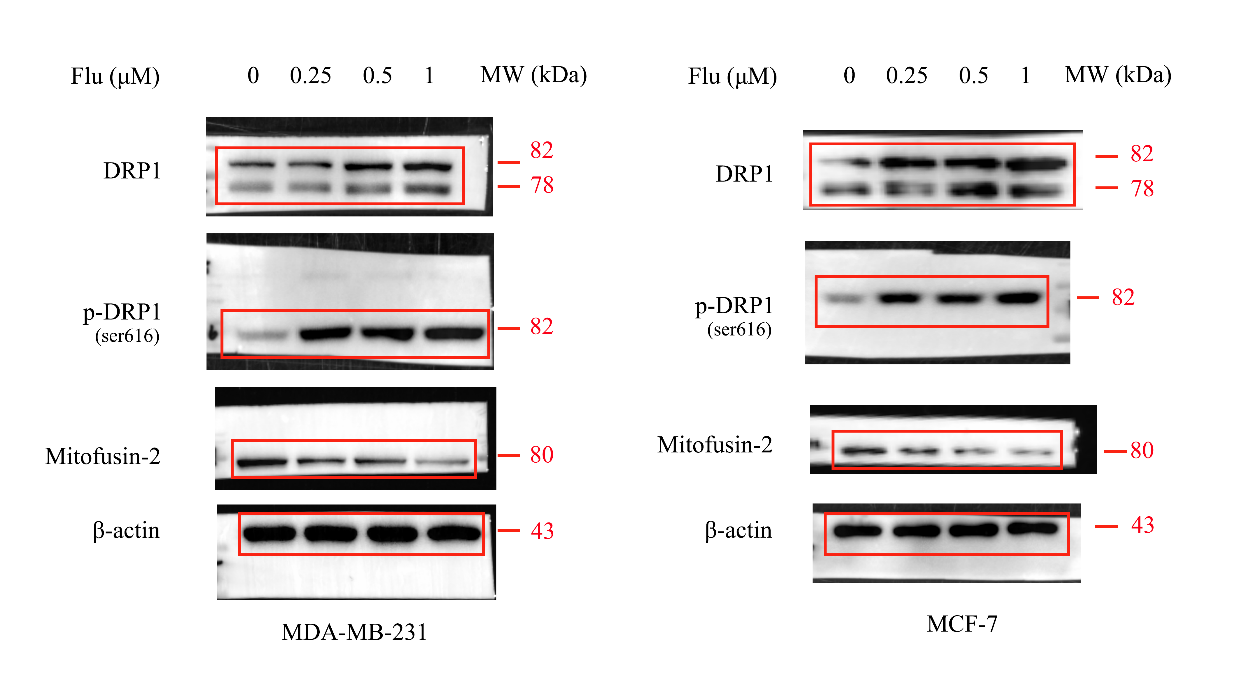


**Figure. 3G**


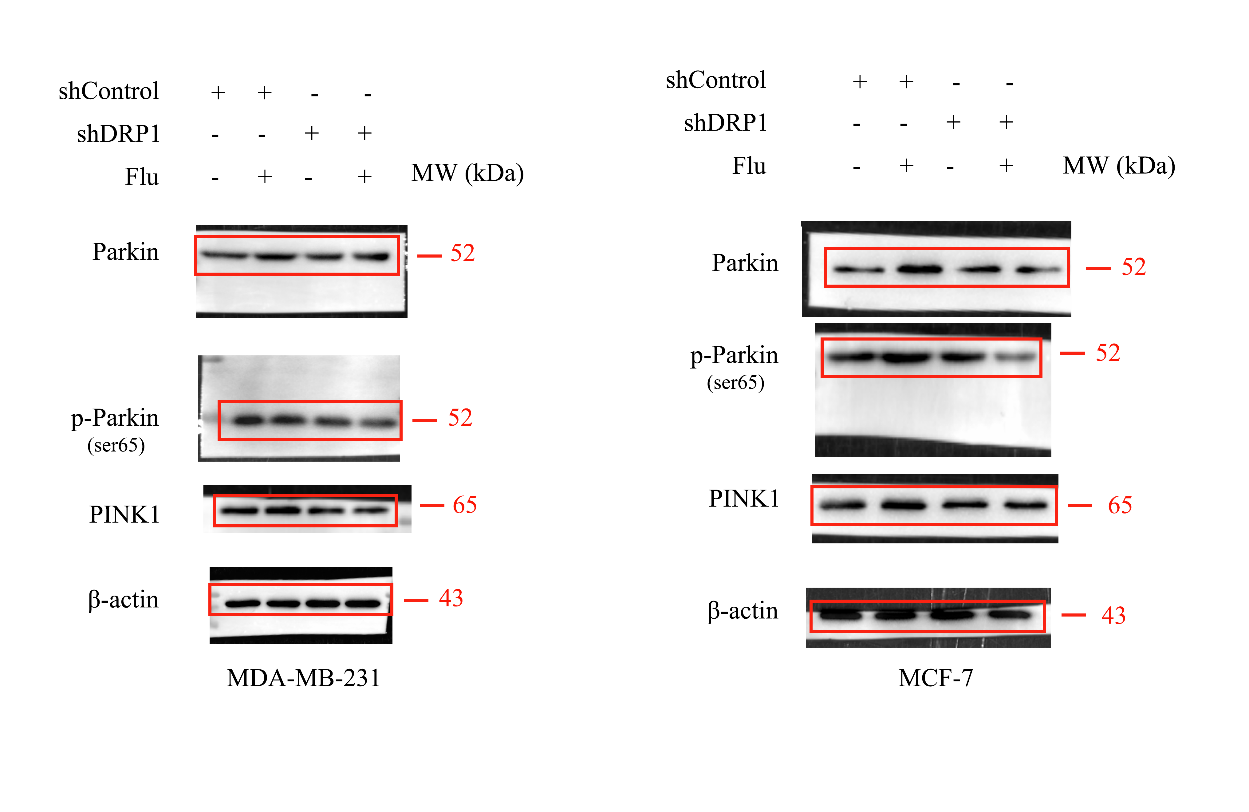


**Figure. 5E**


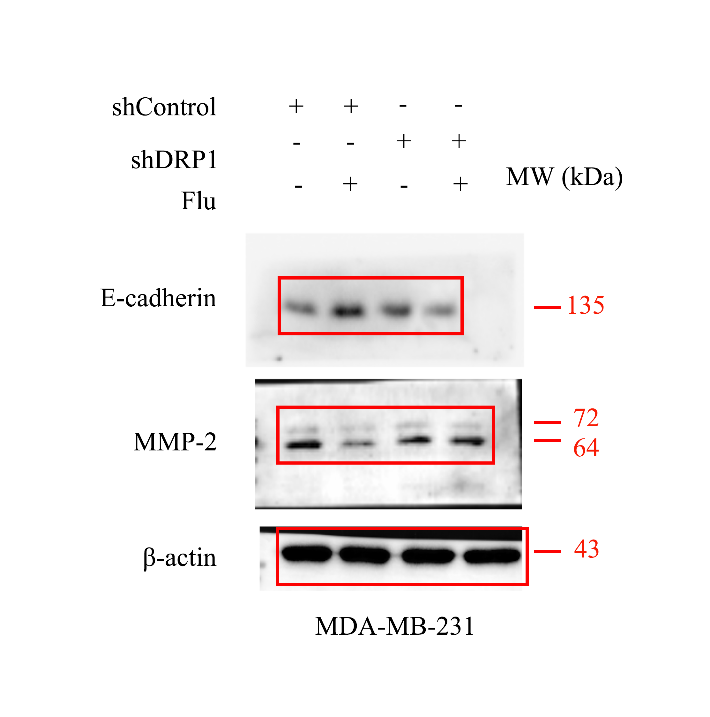


**Figure. 6A**


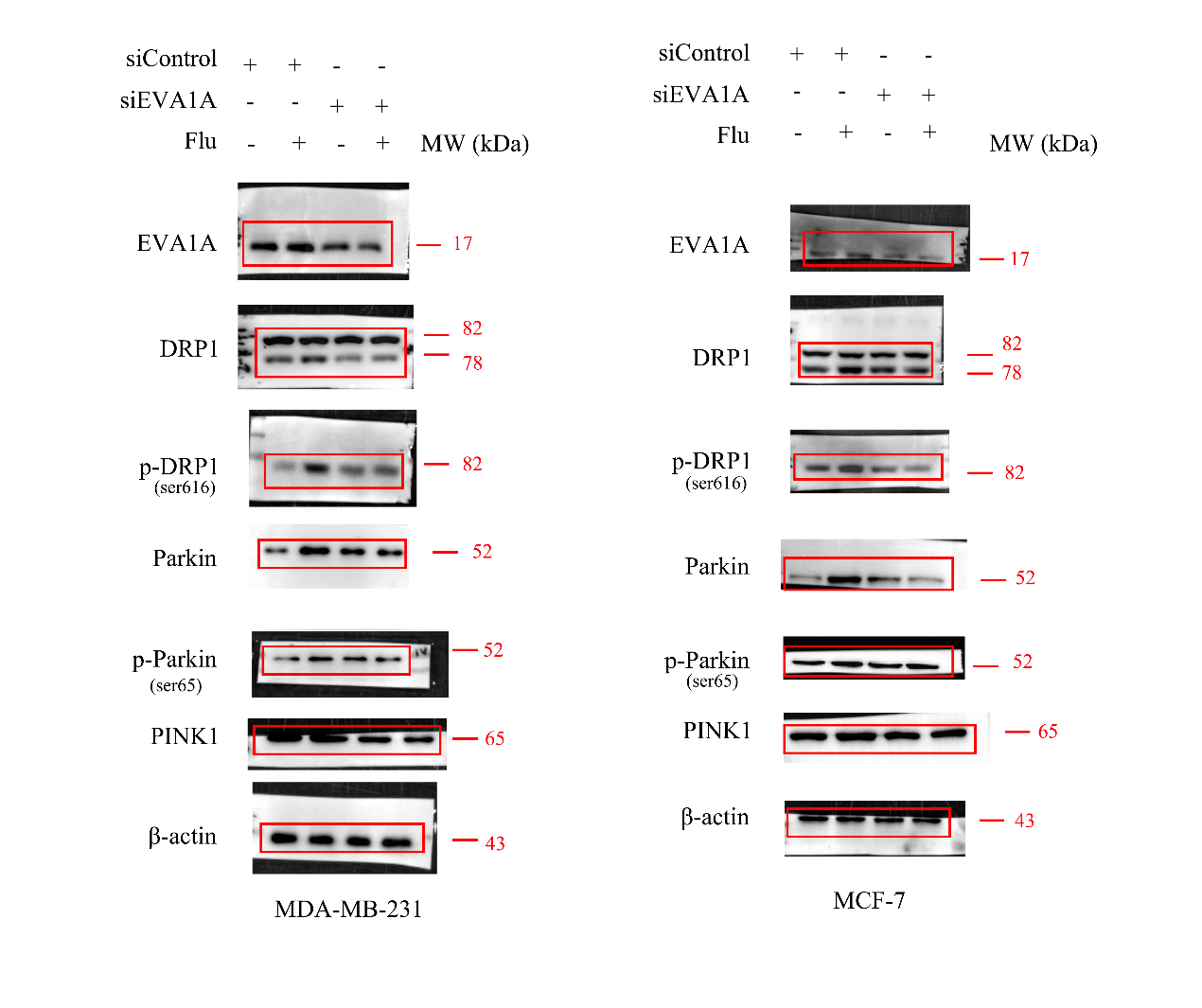


**Figure. 7A**


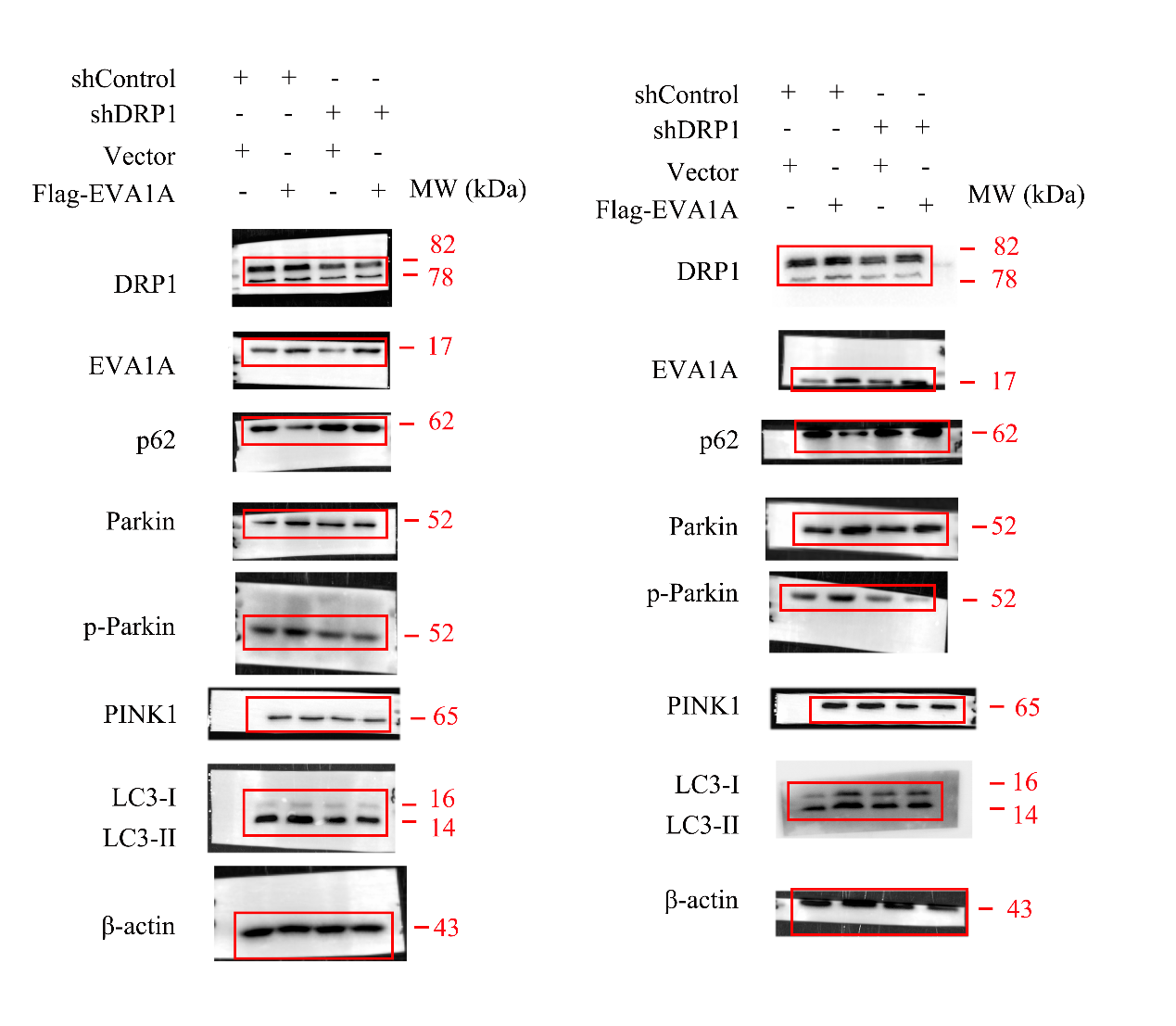


**Figure. 8J**


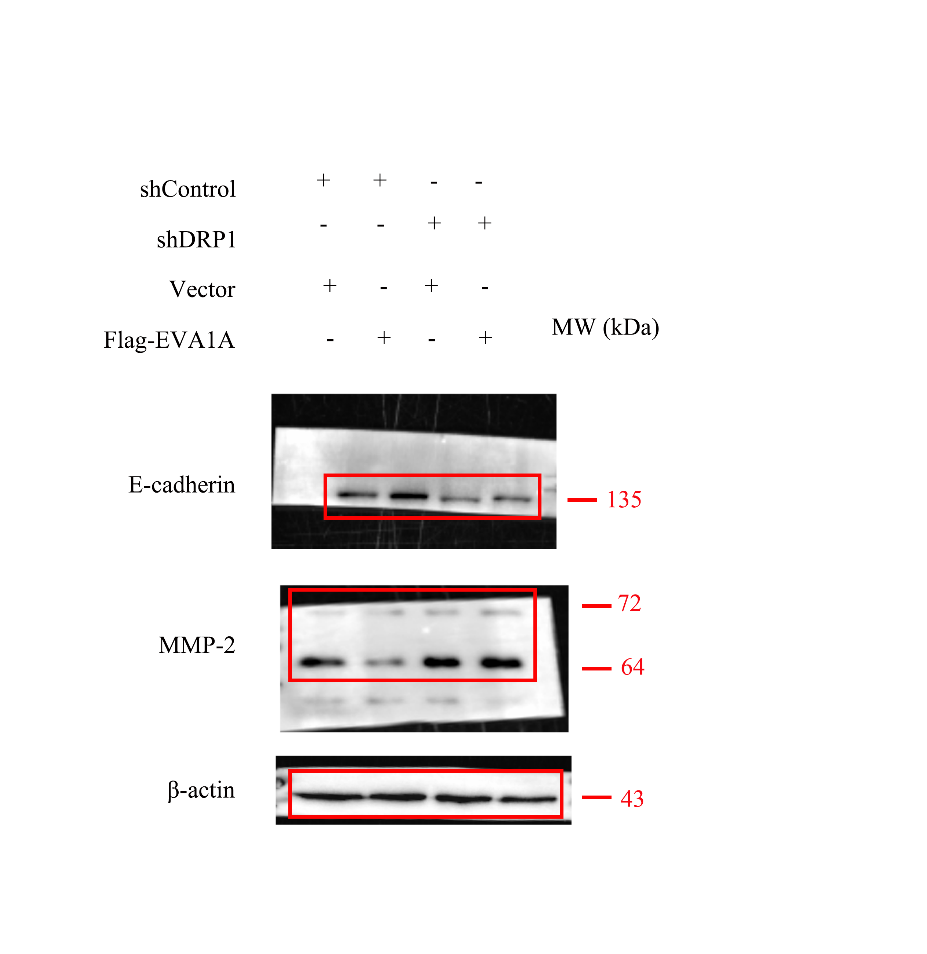


**Supplement figure. 1A**


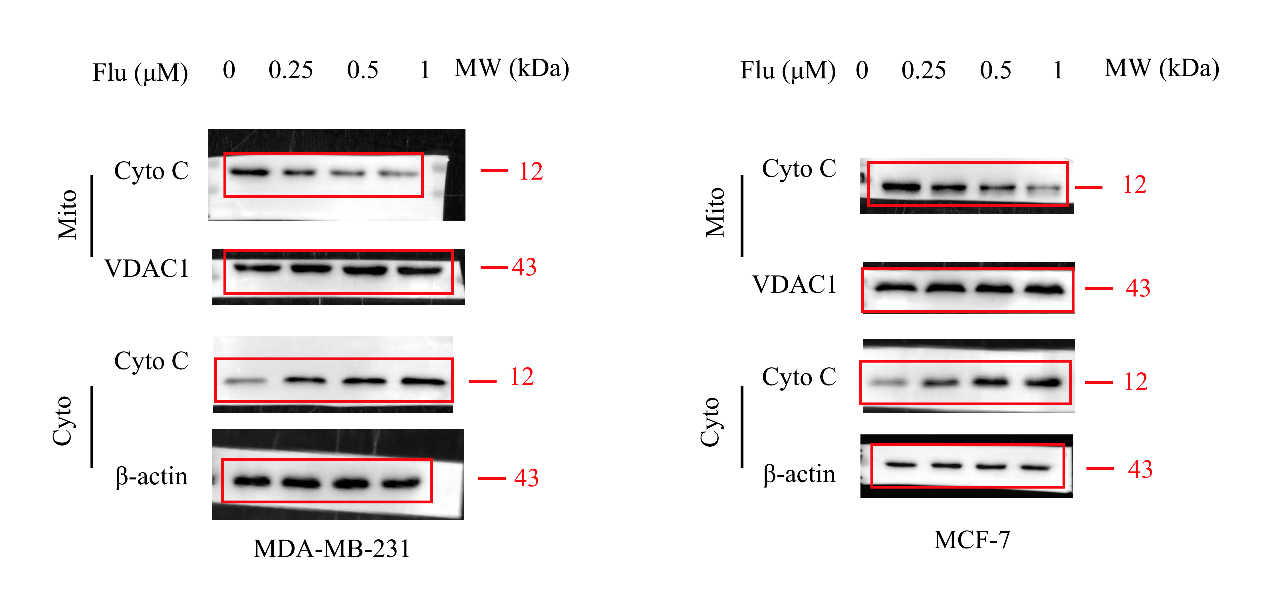


**Supplement figure. 3A**


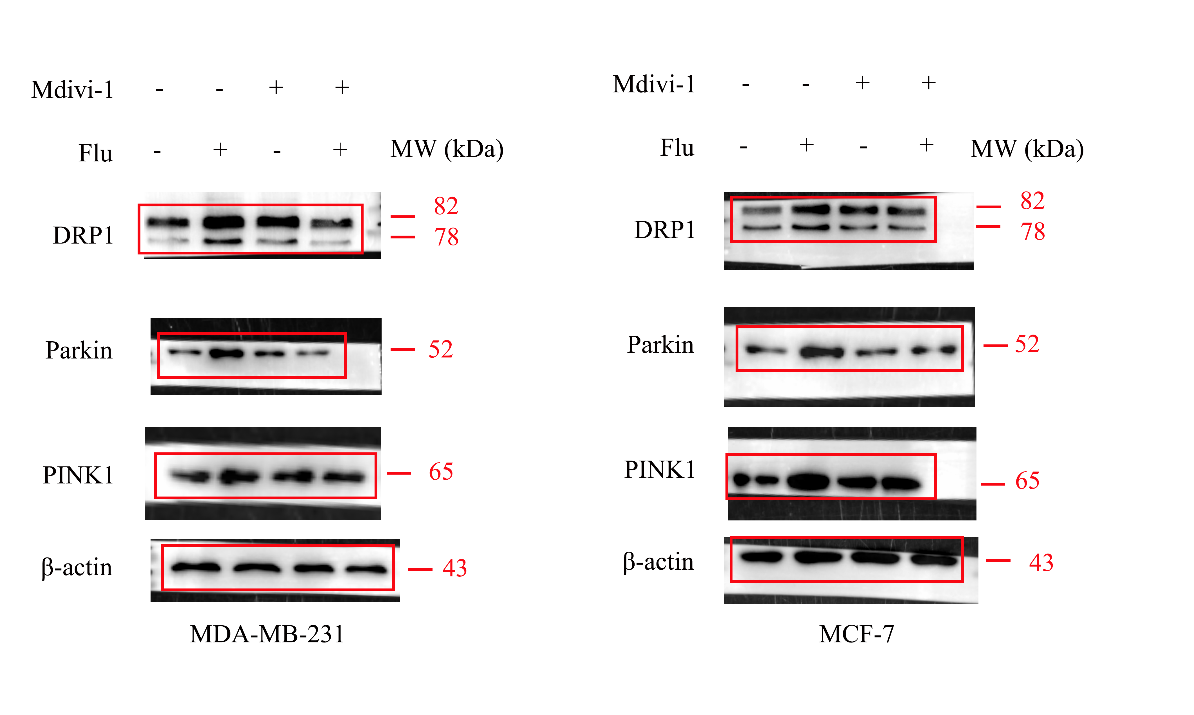


**Supplement figure. 4F**


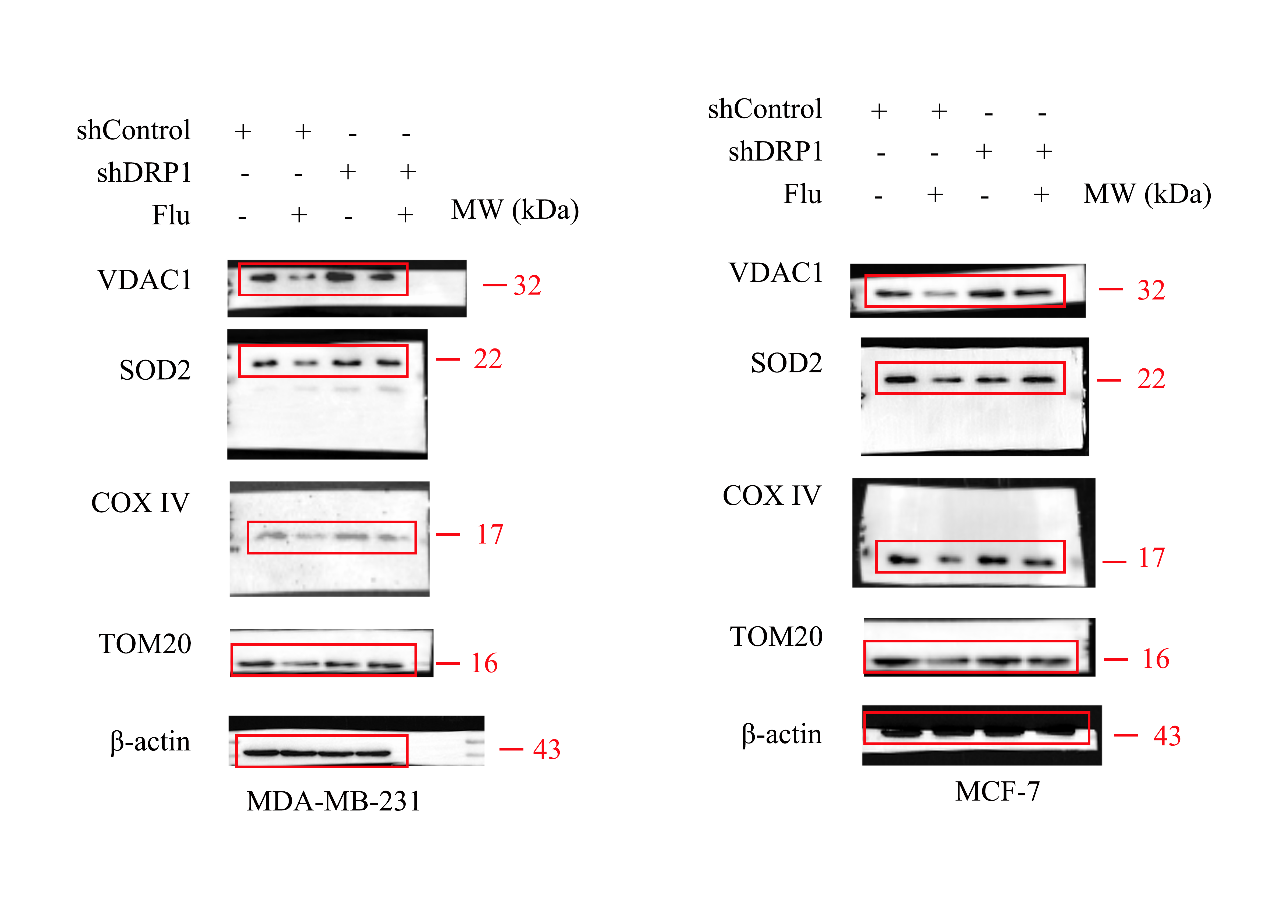

Supplement: Supplementary file 2 — Original Data File [file 41419_2022_4823_MOESM2_ESM.docx]
