# Supplementary material for: Flubendazole induces mitochondrial dysfunction and DRP1-mediated mitophagy by targeting EVA1A in breast cancer
Source: Cell Death Dis. 2022 Apr 19;13(4):375. doi: 10.1038/s41419-022-04823-8 (PMC9019038; doi:10.1038/s41419-022-04823-8)
Supplement: Supplementary file 4 — Author change agreement [file 41419_2022_4823_MOESM4_ESM.pdf]

Author change agreement request

我 2022-03-30 12:44
发至 zhanglanx_9@126.com 、zhouxl 、simplezyq 、2461997888 、2401330343 、910717627 、1143005407 、1624381752

收起

CDDIS-21-2498RR Author changes agreement request

Dear co-authors,

This is an explanation and confirmation message. During the revision process of the manuscript "Flubendazole induces mitochondrial dysfunction and DRP1-mediated mitophagy in breast cancer by targeting EVA1A", Zhaoxin Yuan made great contributions, including collected the materials and analyzed the data. Therefore, we have invited Zhaoxin Yuan as one of our co-authors. Thus, we confirm with all co-authors whether agree with the above decision. Thank you for everything you have done and we look forward to your kind reply.

Best regards,

Dr. Leilei Fu

Response of Dr. Yongqi Zhen

Y YONGQI ZHEN 2022-03-30 12:55
发至 Francie；抄送 我 、zhanglanx_9@126.com 、zhouxl 、2461997888 、2401330343 、1143005407 、1624381752

收起

Re: CDDIS-21-2498RR Author changes agreement request

I have received the message, I agreed that revise.

展开引用

Response of Zhaoxin Yuan

瞻 阿瞻 2022-03-30 13:07
发至 我

收起

回复：CDDIS-21-2498RR Author changes agreement request

Dr. Fu

Thank you very much for your invitation. I agree with your proposal.

Best regards,

Zhaoxin Yuan

展开引用

Response of Jiahui Zhang

2401330343 2022-03-30 13:09
发至 我

收起

Re: CDDIS-21-2498RR Author changes agreement request

I have received the message, I agreed that revise.

展开引用

Response of Yao Chen

陈尧 2022-03-30 12:59
发至 我
收起

回复：CDDIS-21-2498RR Author changes agreement request

I have received the message, I agreed that revise.

展开引用

Response of Yuning Fu

Francie 2022-03-30 12:51
发至 我、zhanglanx_9@126.com、zhouxl、simplezyq、2461997888、2401330343、1143005407、1624381752
收起

回复：CDDIS-21-2498RR Author changes agreement request

I have received the message, I agreed that revise.

Response of Yi Liu

Re: CDDIS-21-2498RR Author changes agreement request leilei_fu@163.com 升级会员

林七月 2022-03-30 13:10
发至 我
详情

I have received the message, I agreed that revise.

---Original---

Response of Dr. Lan Zhang

zhanglanx_9@126.com 2022-03-30 12:47
发至 我
详情

Dear Dr. Fu

I agree with this author change.

Best
Dr. Lan Zhang

Response of Prof. Xian-Li Zhou

周先礼 2022-03-30 14:32
发至 我
详情

I have received the message, I agreed that revise.

Xianli Zhou

展开引用
